# Peculiarities of room temperature organic photodetectors

**DOI:** 10.1038/s41377-025-01939-2

**Published:** 2025-10-09

**Authors:** Antoni Rogalski, Jin Wang, Fang Wang, Zhiping He, Weida Hu, Piotr Martyniuk

**Affiliations:** 1 Institute of Applied Physics, Military University of Technology, 2 Kaliskiego St., 00-908 Warsaw, Poland; 2 State Key Laboratory of Infrared Physics, Shanghai Institute of Technical Physics, Chinese Academy of Sciences, 500 Yu Tian Road, Shanghai, 200083 China

**Keywords:** Photonic devices, Nanophotonics and plasmonics

## Abstract

Organic semiconductors (OSCs) have been considered as projecting family of optoelectronic materials broadly investigated for more than 40 years due to capability to tune properties by adjusting chemical structure and simple *processing*. The OSCs performance has been substantially increased, due to the fast development in design and synthesis. The spectral response of OSCs was extended from ultraviolet (UV) to near infrared (NIR) wavelength region. There are papers reporting detectivity (D^*^) higher than the physical limits set by signal fluctuations and background radiation. This paper attempts to explain the organic photodetectors’ peculiarities when confronted with typical devices dominating the commercial market. To achieve this goal, the paper first briefly describes OSC deposition techniques, diametrically opposed to those used for standard semiconductors. This was followed by a more detailed discussion of basic physical properties, contributing to the photodetectors’ performance including absorption coefficient, conduction mechanism, charge generation and charge transport. These effects are very different from those found in inorganic semiconductors (ISCs). The second part of the paper describes the main modes of OSC based photodetectors [photoconductors, photodiodes and field effect transistor photodetectors (FET)] with emphasis on their special features that distinguish them from standard photodetectors. Final part of the paper shows current state-of-the-art of various types/structures of photodetectors and routes for further improvement. The upper detection limit for OSC photodiodes has been shown to be comparable to that for ISC photodiodes with nearly three orders of magnitude variation. The D^*^ overestimates (especially organic based FET phototransistors) were explained.

## Introduction

Organic semiconductors (OSCs) have been investigated for more than forty years with the capabilities to renovate important technologies, to include photovoltaic energy, transparent screens/displays, effective and rationally cost white lighting or robust and flexible electronics^1–10^, however display/lighting development have been of great interest. The exceptional properties make OSCs appropriate for detectors operating from ultraviolet (UV) to near infrared (NIR) with specific wavelengths tuning capability^6–8,10^. Extensive research on OSCs have been carried out on exploring materials, device structures, physical mechanisms, and processing approaches to improve the performance of organic photodetectors to the level of their ISCs counterparts such as Ge, GaN, GaAs or InGaAs.

Compared to ISCs, OSCs offer several distinct advantages:

- OSCs are normally low-cost and matching with large area (low temperature and cheap production methods matching with high-yield roll-to-roll *processing*),
- OSCs based devices are fabricated on the plastic films, metal foils or glass (lattice mismatch tolerance and deformation-induced defect states, majority of inorganic materials require high quality substrates),
- organic optoelectronic materials possess the ability to adjust their optical bandgap through chemical modifications of their molecular structure [this enables UV, visible (VIS), or NIR detection without the need for filters, thereby simplifying the spectral selectivity of detectors],
- the mechanical conformability of organic materials inherently provides advantages over inorganic materials in the development of large-area, flexible, wearable photodetectors making them well-suited for diverse and evolving future needs,
- narrowband OSCs exhibit capabilities for both colour-selective and panchromatic detection, offering significant application advantages in medical imaging compared to inorganic photodetectors.

Even though several advantages, OSCs also have disadvantages. One of the primary ones is the low carriers’ mobility related to the weak intermolecular interactions reducing performance compared to inorganic devices. In addition, majority of organic materials are found not to be very stable, being susceptible to degradation by water vapour and oxygen exposure requiring special housing to reach the satisfactory device durability. Another problem is related to the organic materials purity—much lower than inorganic materials, with the consequent creation of electronic defects reducing device performance.

This article consists of three parts. In the first one the fundamental physical properties of the organic materials including conduction mechanism, charge generation and transport are discussed. Also, their deposition techniques are shortly described. The second part explains operation principles of different types of organic photodetectors. The last part of the paper presents status of organic photodetectors along with predictions for further improvements.

A number of abbreviations for organic materials names were introduced in the paper. Their explanation is made in Table 1.

**Table 1:** List of abbreviations for organic materials

Abbreviation	Meaning
OSC	Organic semiconductor
ISC	Inorganic semiconductor
HOMO	Highest occupied molecular orbital
LUMO	Lowest unoccupied molecular orbital
SOMO	Singly occupied molecular orbital
OLED	Organic light-emitting diode
OPD	Organic photodiode
BHJ	Bulk heterojunction
q-PHJ	Quasi-planar heterojunction
PEDOT:PSS	poly(3,4-ethylene dioxythiophene): polystyrene sulfonate
FBR	5,5′-[(9,9-Dioctyl-9*H*-fluorene-2,7-diyl)bis(2,1,3-benzothiadiazole-7,4-diylmethylidyne)]bis[3-ethyl-2-thioxo-4-thiazolidinone]
IDTBR	(5Z,5’Z)-5,5’-((7,7’-(4,4,9,9-tetraoctyl-4,9-dihydro-s-indaceno[1,2-b:5,6-b’]dithiophene-2,7-diyl)bis(benzo[c][1,2,5]thiadiazole-7,4-diyl))bis(methanylylidene))bis(3-ethyl-2-thioxothiazolidin-4-one)
PTB7-Th	poly[4,8-bis(5-(2-ethylhexyl)thiophen-2-yl)benzo[1,2-*b*;4,5-*b*′]dithiophene-2,6-diyl-alt-(4-(2-ethylhexyl)−3-fluorothieno[3,4-*b*]thiophene-)−2-carboxylate-2-6-diyl)]
PBDBT-DTBT	([[4,8-bis[5-(2-ethylhexyl)−4-chloro-2-thienyl] benzo[1,2-*b*:4,5-*b*’]dithiophene-2,6-diyl]−2,5-thiophenediyl[5,7-bis(2-ethylhexyl)−4,8-dioxo-4*H*,8*H*-benzo [1,2-*c*:4,5-*c*’]dithiophene-1,3-diyl]−2,5-thiophenediyl])
IDTBT	polymer of 2,1,3-Benzothiadiazole-4,7-diyl-co-4,4,9,9-tetrahexadecyl-4,9-dihydro-*s*-indaceno[1,2-*b*:5,6-*b*‘]dithiophene-2,7-diyl
DPPTTT	[poly-[2,5-bis(2-octyldodecyl)-3,6-di(thiophen-2- yl)pyrrolo [3,4-c]pyrrole-1,4(2H,5H)-dionel-alt-thieno [3,2-b]thiophene]
PSeDPPBT	poly(3,6-bis-selenophene-2-octyl-1-dodecyl-diketopyrrolopyrrole-co-benzothiadiazole
PBTTT	poly[2,5-bis(3-tetradecylthiophen-2-yl)thieno[3,2-b]thiophene]
P3HT	poly(3-hexylthiophene)
PC_71_BM	[6,6]-phenyl C71 butyric acid methyl
PC61BM	[6,6]-phenyl-C61-butyric acid methyl
PDPDBD	poly dithienobenzodithiophene-co-diketopyrrolopyrrolebithiophene
TPD	poly(N,N’-bis-4-butylphenyl-N,N’-bisphenyl)benzidine
ITIC	3,9-bis(2-methylene-(3-(1,1-dicyanomethylene)-indanone))-5,5,11,11-tetrakis(4-hexylphenyl)-dithieno[2,3-d:2’,3’-d’]-s-indaceno[1,2-b:5,6-b’]dithiophene
POT-12	poly (3,3‴-didodecyl quarter thiophene
F4-TCNQ	2,3,5,6-tetrafluoro-7,7,8,8- tetracyanoquinodimethane
NDI	naphthalene diimide
PM	phenylmethyl
HATNA-Cl_6_	2,3,8,9,14,15-hexafluoro-5,6,11,12,17,18-hexaaza-trinaphthylene
W_2_(hpp)_4_	tetrakis(1,3,4,6,7,8-hexahydro-2H-pyra[pyrimidinato)ditungsten(II)

## The organic semiconductors fundamentals

**Fig. 1:**
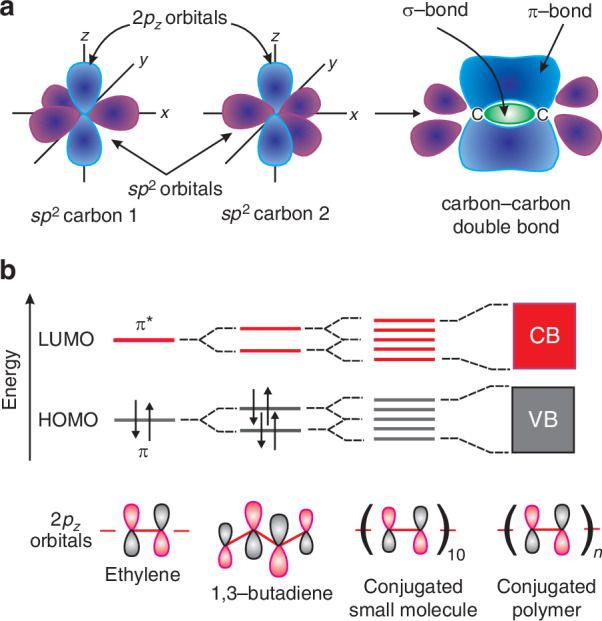
Carbon (sixth element in the periodic table, electron configuration 1s^2^2s^2^2p^2^) as the main building block of OSCs. **a** The combination of the sp^2^ (hybridized) and 2p_z_ (unhybridized) orbitals to create the σ-/π-bonds in a carbon-carbon double bond. **b** Origin of OSC’s band structures - extension of molecular orbitals to long conjugated oligomers and polymers (Reproduced with permission for ref.^11^ © 2020 Griffith, Cottam, Stamenkovic, Posar and Petasecca). CB-conduction band, VB-valence band

OSCs build the group of the carbon-based compounds - they may also contain other organic elements such as hydrogen or oxygen. Carbon (sixth element in the periodic table, electron configuration 1*s*^2^2 *s*^2^2*p*^2^) as the main building block decisively influences the properties of OSCs. The electron configuration suggests that the orbitals of the first and second shells are fully occupied by two electrons. The other two electrons are distributed on two of the three degenerate *p*-orbitals accommodating a maximum of six electrons. Adjacent carbon atoms in the OSC molecule form chemical bonds while a common electron density is located on the hybrid orbitals shaped by interference between the 2*s* and 2*p* orbitals [see Fig. 1a]^11^. In the case of the bonds between two carbon atoms, single atom has three neighbours where one electron origins from the *s*-orbitals and two from the *p*-orbitals forming three new hybridized *sp*^2^ orbitals adopting the equilateral triangle geometry. The remaining carbon *p* orbital (2*p*_*z*_) is not hybridized with axis perpendicular to the hybridized *sp*^2^ orbitals. As is shown in Fig. 1a, the carbon atoms are connected by three σ bonds by sharing electrons on the *sp*^2^ orbitals and a single *π*-bond by sharing electrons on the 2*p*_*z*_ orbitals.

Carbon atoms form molecules with other carbon or non-carbon atoms and their electronic wavefunctions are combined into molecular wavefunctions, while electrons from each atom are split between new molecular orbitals. This process is shown in Fig. 1b with a *π*-conjugated network built of increasing number of carbon atoms^12^. The 2*p*_*z*_ orbitals overlap (linear superposition) to create a single molecular orbital where electron density is delocalized across the molecule. This mechanism can be qualitatively described by the molecular orbital approximation (linear combination of atomic orbitals).

The *π*-electrons drives the OSCs electronic characteristics. As the OSC’s conjugation length increases, the closely spaced energy levels form the “band” structure like that in inorganic semiconductors. Analogous to the latter, the valence band (VB) in OSC is the highest occupied molecular orbital (HOMO, or *π*-orbital), and similarly - the conduction band (CB) is the lowest unoccupied molecular orbital (LUMO, or π*-orbital). Unlike in ISCs (free charge carriers), the OSCs photo-excited state occurs as a bound electron-hole pair (exciton).

**Fig. 2:**
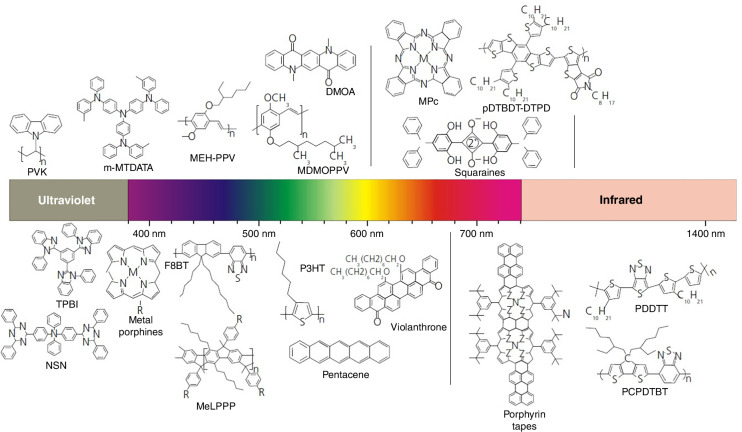
Example of donor molecules for OSC photodetectors for UV to the short wavelength infrared (SWIR)

OSCs may be categorized as single small molecules, oligomers (few monomer units) and polymers (many monomer units). The OSCs are characterized by the relatively low energy (≈10 kcal/mol) van der Waals (vdW) intermolecular bonds compared to e.g. Si-Si (78 kcal/mol) covalent bonds. This means that the propensity to form ordered structures is mild, however from the OSCs fabrication point of view, this is advantageous. Many OSCs may be functionalized by diluting them in proper solvents enabling deposition by adapting low-cost solution-based techniques. Assuming the deposition methods and type of molecule, aggregation and packing in the solid state may result in fabrication (i) amorphous materials, (ii) heterogeneous materials with nano- and microcrystalline regions interspersed in the amorphous matrix, and even (iii) monocrystalline materials. Figure 2 presents the most widely fabricated *π*-conjugated molecules covering UV−NIR range.

Table 2 describes the OSCs advantages and disadvantages. The required *processing* techniques were reported to be one of the clearest differences between ISCs and OSCs. ISCs are fabricated on expensive, highly pure/quality substrates, high temperature condition and low throughput techniques, while OSCs may be deposited on inexpensive substrates to include glass, plastic, metal foil and due to the molecular nature, the solution-based methods may be implemented. Most of the OSCs small molecules are cleaned by multiple gradient zone sublimation.

**Table 2:** Pros and cons of organic semiconductors

Advantages	Disadvantages
• Mechanical flexibility – deposited onto flexible substrates – creation of bendable and foldable electronic devices • Fabrication by the solution-processed – techniques like printing, significantly reducing manufacturing costs	• Relatively low carrier mobilities – particularly challenging for high-speed electronics applications • Low stability – susceptible to environment conditions: temperature, humidity and oxygen • Grain boundaries – disrupt the charge transport and affect device performance

The most prevalent deposition techniques for OSC thin films are vacuum evaporation and spin coating. Vacuum evaporation boasts a significant advantage in preserving the purity of OSC by preventing modification from water and oxygen. Furthermore, it allows for OSC deposition at lower temperatures, which can significantly cut costs and facilitate wafer-level OSC preparation. In contrast, the spin coating method for OSC preparation is characterized by its straightforward process and low cost, enabling the creation of thick OSC thin films. Table 3 compares these techniques with other promising small molecules and polymer methods for industrial OSCs electronic manufacturing.

**Table 3:** OSCs deposition techniques

Techniques	Operation	Characteristics
**Vacuum thermal evaporation**	The system is built of a vacuum chamber typically reaching ~10^-6^ mbar containing several thermal sources (temperature controlled). To avoid uncontrolled deposition, the chamber is equipped with proper shutters to protect the samples and to isolate sources. The ultrahigh vacuum systems (pressure < 10^−9^ Torr) are to deposit small molecules in molecular beam deposition.	Method is used to deposit OSC that can resist the sublimation/evaporation without degradation (e.g. small molecules and oligomers). One benefit is the ability to achieve nanometer-scale control of multilayer stacking and high uniformity over a large area. Vacuum thermal evaporation also allows patterning of photodetectors with masks (~100 μm with chemically etched masks). Higher resolution may be reached by laser patterning. The technique is a favourite path for doping. The rates of co-evaporated materials may be accurately measured and monitored.
**Spin-coating**	This technique is undoubtedly the most extensively used for solution-based OSCs deposition. The best-known industrial application is photolithography in ISC microelectronics. The solvent solution is dispensed onto the substrate being rotated. The drying step is to remove residual solvent from the films.	The uniform layers with controlled thicknesses (from tens of nanometres to microns) may be deposited, however, does not allow for lateral patterning requiring further *processing*. The realization of large area detector arrays and scalability are limited, although the technique is superb for laboratory research. Technique is material consuming (dispensed solvent onto the substrate is lost during the initial spin-off).
**Ink-jet printing**	Injection printing is divided into two categories: continuous injection printing (CIJ) and on-demand droplet printing (DOD) based on droplet direction. In CIJ, droplets are formed by a transducer or loading apparatus that produces a continuous stream. The electrostatically charged droplets are deflected by an electric field according to the pattern reaching the substrate or being recirculated via gutter. The DOD is simpler due to the more effective source usage (droplet produced when needed). Piezoelectric transduction is most often preferred for functional inks because it can be applied to a vast set of formulations.	Inkjet printing is a very flexible technique being resistant to the conventional organic materials solvents, insulators and metallic inks. The controlled volume of ink at specific places on the substrate can be deposited being suitable for patterning inks to process optoelectronic device arrays, in a direct writing approach (reduced material waste, no masks needed). The CIJ technique is used for low-precision industrial applications (e.g. packages/goods labelling) but is not flexible enough for printed electronics. Instead, DOD’s approach is most used for functional electronics research.
**Aerosol-jet printing**	Aerosol printing is like inkjet printing. The ink is sprayed by ultrasonic/pneumatic atomizer and moved to the print head by a carrier gas. The aerosol flow diameter is limited by the carrier gas, occupying the nozzle outer layer. The final jet flow size results from nozzle diameter, sheath gas and aerosol flow rates.	Aerosol-jet printing is a new type of incremental fabrication technology with industrialization potential. This non-contact, programmable and versatile printing technique can reach a ~10 μm resolution. Useful for materials with a very wide viscosity range.
**Spray-coating**	Spray may be created by the pneumatic systems (pressurized gas), ultrasonic systems and electro-spray systems. The system consists of a gun with an attached nozzle into which an ink mist generated by the gas/air jet is fed. The patterns depend significantly on the spray particles size/distribution and coalescence on the substrate. The decisive quality parameters of the atomization process are the ink surface tension and viscosity related to the selected spraying scheme.	Spraying may be considered as the simple version of aerosol jetting (reduced cost, atomized ink less focused). Spraying allows large area coatings being compatible with roll-to-roll printing lines. The main challenge is uniformity and high-quality films (micrometre-diameter solution droplets deposited on the substrate).

Over the past 20 years, also a variety of chemical deposition techniques have been employed to produce nanostructured thin films. One of the most popular is the chemical vapor deposition (CVD), which provides unique advantages in thin film deposition, including high-purity, uniformity, and scalability. Its ability to deposit a wide range of materials and control film properties makes it invaluable in industries like electronics, aerospace, and manufacturing. However, its high operating temperatures, limitations in coating large surfaces, logistical challenges (higher costs), are notable drawbacks.

The physical properties of OSCs differ significantly from their ISCs counterparts. In the former, the strong electron-phonon coupling along with the lack of long-range ordering results in the localization of charge carriers on a single molecule or few adjacent molecules (to compare—the ISCs Bloch wave functions are considerably fuzzy). The consequence of this is relatively inefficient transport of charge carriers by tunnelling among molecules (*hopping*). In addition, the high Coulombic interaction among carriers and the strong polaron coupling among the localized *hopping* states needs to be taken into consideration. Furthermore, the molecules of OSC depend on vdW force interactions, and the delocalization of their charge carriers takes place within a confined range, leading to discrete and relatively narrow energy bands. The weak bonds in OSCs make the intermolecular spacing between atoms and carriers’ mean scattering length comparable meaning that charge transport cannot be described by the band transport theory applied to ISCs^13,14^. On the other hand, however, such carrier localization makes these OSCs more tolerant to impurities than ISC counterparts, making the deposition techniques simpler and reducing *processing* costs.

In-depth discussions of the various processes and doping methods were reported in several review papers^15–17^. Generally, OSCs doping is limited to few small molecules and is not yet well mastered exhibiting the following issues: the dopant molecule environmental stability, dopant ionization effectiveness (related to the relatively low OSCs dielectric constant *ε*_*r*_ ≅ 3 − 4) and thermodynamic dopants stability related to interstitial nature. Molecularly doped polymers may be considered as an exception to this rule. An example is poly(3,4-ethylendioxythiophene) polystyrene sulfonate SOMO, where the semiconducting polymer PEDOT is p-doped by PSS sulfonyl groups. PEDOT:PSS is very stable with conductivity reaching the level 100–1000 S/cm.

**Fig. 3:**
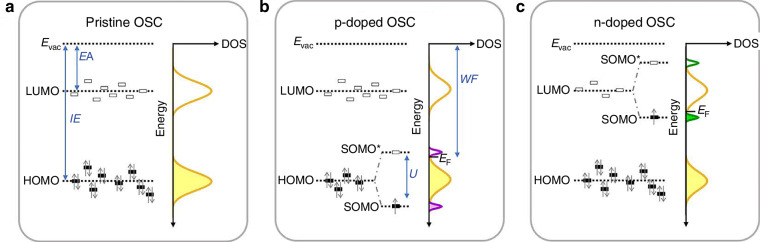
The OSCs doping fundamental principles. **a** Pristine. **b** p-doped. **c** n-doped OSCs. The energy levels (occupied bands are shaded, and empty bands are clear) and corresponding density of states (DOS). U gap is created between the SOMO and SOMO* bands of the doped OSCs. The doped OSC work function (WF) is a convolution of the shallowest occupied and deepest empty states. IE-ionization energy, EA-electron affinity (Reproduced with permission for ref.^15^ © 2023 Tang, Hou, Leong. Published by American Chemical Society)

The p- and n-dopants [characterized by large electron affinities (EAs) and small ionization energies (IEs)] are introduced into the OSC by the solution mixing, vapor deposition, sequential doping and thermal evaporation. Figure 3 presents the OSCs doping’s fundamental principles. In terms of ISCs the donors or acceptors are added to the material generating additional charge carriers. To reach n-type OSC, the dopant must donate electrons to the LUMO while to create holes the p-type dopants take electrons from HOMO. Electrons transferred from the OSC to the p-dopant generate the hole polaron. The hole polaron’s singly occupied molecular orbital (SOMO) is derived from the splitting of the HOMO into a SOMO and an empty SOMO^*^ counterpart [separated by the gap (*U*) due to electron-electron interactions in the SOMO]. The Coulomb interactions among counter ions and holes further shifts SOMO downwards in relation to the HOMO [Fig. 3b]. Correspondingly, the n-doped OSC creates an electron polaron and the SOMO band appears below and the empty SOMO^*^ band above the LUMO localizing a discrete electron polaron state in the gap [Fig. 3c].

**Fig. 4:**
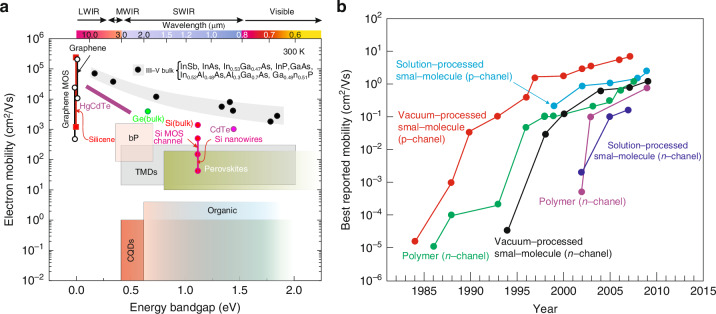
Electron mobilities at room temperature. **a** Comparison of mobilities of various material systems with standard semiconductors used in the photodetectors’ fabrication [transition metal dichalcogenides (TMDs), colloidal quantum dots (CQDs), black phosphorus (bP)]. **b** The field dependent mobility evolution in organic FET transistors (Reproduced with permission for ref.^21^ © American Chemical Society)

The carrier mobility is low, normally within 10 ^− 4^− 10^0 ^cm^2^/Vs range, and is considerably lower than in conventional inorganic semiconductors — see Fig. 4(a). The range of mobility changes for different materials is highlighted from refs. ^18–20^; and for organic materials from refs. ^21,22^.

The carrier mobilities in the range of tens of cm^2^/Vs were reported in the latest generation of high-performance polymers (only for high carrier densities ~10^19 ^cm ^− 3^)^23^. Such high carrier densities were reported in the transistors’ accumulation channel. The results shown are not reproducible very well and depend on device *processing* technology. Figure 4b shows the time evolution of carrier mobilities in organic FET transistors. The field dependent mobility is much larger than that of amorphous silicon (0.5 − 1.0 cm^2^/Vs) reaching ≥10 cm^2^/Vs. The advances in mobility offer great potential in OSCs based devices applications: such as organic CMOS circuits for radio frequency identifications and light-emitting diodes (OLEDs).

**Fig. 5:**
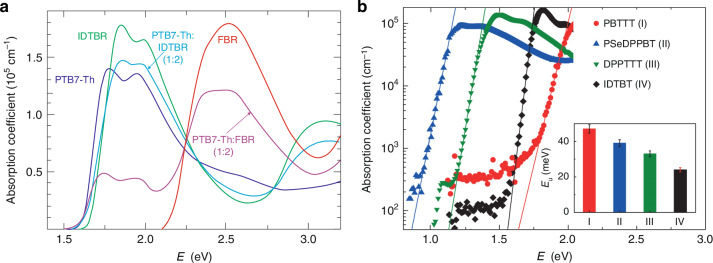
Absorption coefficient of organic molecules. **a** Non-fullerene acceptors FBR 27 (red) and IDTBR28 (cyan), donor polymer PTB7-Th29,30 (blue), and their blends (ratio 1:2) (purple, light blue) extracted from UV − VIS measurements (Reproduced with permission for ref.^23^ © 2018 Krückemeier, Kaienburg, Flohre, Bittkau, Zonno, Krogmeier, Kirchartz). **b** DPPTTT, IDTBT, PSeDPPBT and PBTTT films, measured by photothermal deflection spectroscopy (Reproduced with permission for ref.^24^ © Nature)

**Fig. 6:**
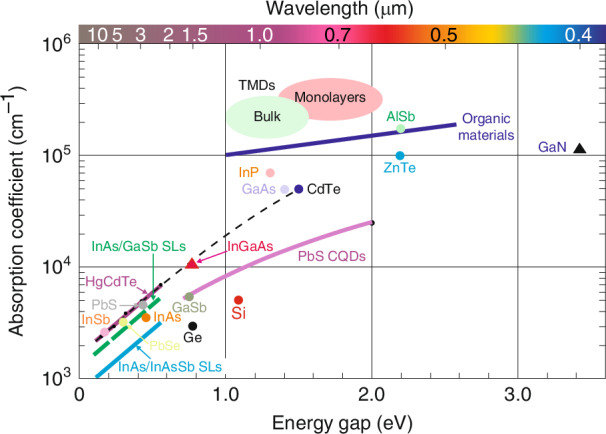
Absorption coefficient at room temperature as a function of the band gap energy for selected materials (Reproduced with permission for ref. ^18^ © 2023 Rogalski, Kopytko, Hu, Martyniuk. Licensee MDPI, Basel, Switzerland)

The OSCs are characterized by high absorption coefficient. Typical *α* assumes 10^4 ^− 10^5 ^cm^−1^, leading to absorption lengths (~1/*α*) ~100 nm. Figure 5 shows the absorption coefficients for selected photovoltaic materials^24,25^. In contrast to ISCs, OSCs exhibit a high absorption coefficient only in a reduced spectral region (typical width of about ~0.5 eV) what is presented in Fig. 5a for non-fullerene acceptors FBR and IDTBR, the donor polymer PTB7-Th 29,30, and their blends. The absorption peak is asymmetric—the rising edge (lower energy range) is steeper than decay counterpart (higher energy). The solid lines, presented in Fig. 5b, represent exponential tail fitting for selected *E*_*u*_ - Urbach energies used to quantify energetic disorder in the band edges of a semiconductor (inset). Absorption coefficient versus energy may be given by the relation $\alpha \left(E\right)={\alpha }_{0}\exp \left[\left(E-{E}_{1}\right)/{E}_{u}\right]$, where ${\alpha }_{0}$ and ${E}_{1}$ are fitting parameters with dimensions of inverse length and energy, respectively. Compared to organic materials, only transition metal dichalcogenides (TMDs) have higher absorption coefficients in the visible range (Fig. 6).

**Fig. 7:**
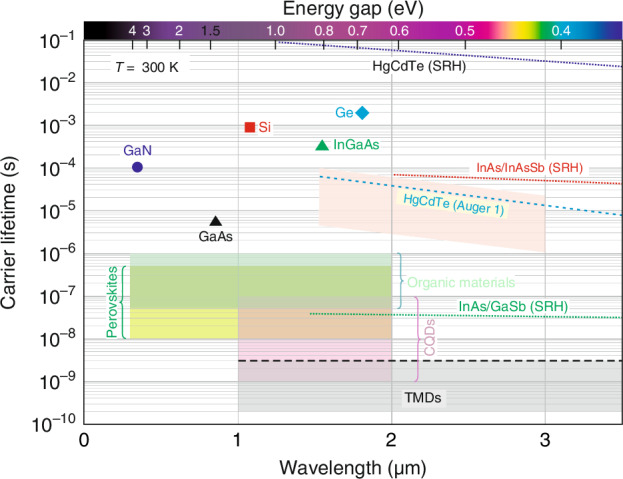
Carrier lifetimes for various material systems

In addition to carrier mobility and absorption coefficient, a major influence on the performance of photodetectors is the carrier lifetime, which affects both the responsivity and the response speed of the detector. As is shown in ref. ^18^, the square root of the absorption coefficient, *α*, of the material and the carrier lifetime, *τ*, ($\sqrt{\alpha \tau }$) can be used as a criterion for the quality of the photodetectors. In general, for thin organic photodetectors the lifetime of carriers decreases as the power of incident radiation increases (this topic is discussed further in “Operating mechanisms of OSC photodetectors”). For this reason, reliable lifetime measurements are conducted under low-power conditions of incident radiation. Figure 7 shows typical values for various material systems^18,19,25^; for organic materials and perovskites from refs. ^20^ and ^26^, respectively. As shown, carrier lifetimes in organic materials are at least two orders of magnitude less than in conventional Ge, Si and InGaAs semiconductors.

**Fig. 8:**
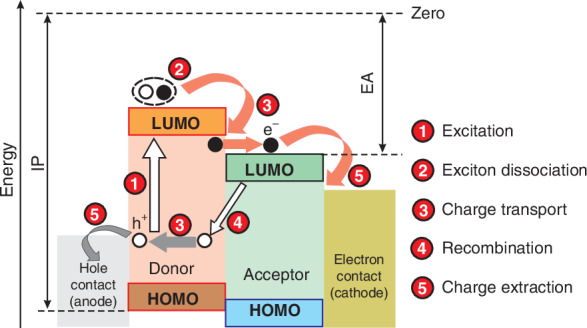
The carriers’ generation mechanisms in OSCs: photoexcitation, exciton dissociation, charge carrier transport, and recombination and extraction processes required to extract free charge

Figure 8 presents the fundamental carrier mechanisms for standard donor/acceptor (D/A) system. There are five primary sequential processes:

- Carrier excitation (exciton generation),
- Exciton dissociation,
- Charger transfer,
- Carrier recombination, and
- Charge extraction.

After absorption, the spinless/neutral excited state is created constituting a Frenkel-type exciton, reassembling electron-hole (e/h) pair with a high (several hundred meV) binding energy. Spontaneous separation of the exciton into a free e/h pair is less effective compared to ISC due to the low potential barrier between the exciton and the charge pair state. The photoinduced charge separation occurs between D/A interfaces by the low donor ionization potential (IP) and the high acceptor EA.

Due to optical excitation in the D/A region, an exciton reaching the interface decays transferring the charge - electrons residing on LUMO (acceptor) and hole residing on the HOMO (donor). To make that process efficient, the required energy difference at the level 0.3 − 0.4 eV between the donor’s HOMO and the acceptor’s LUMO is needed^27^. The photoinduced carriers’ separation occur in the time <100 fs. To achieve high photogeneration efficiency, that process should occur at a distance <diffusion length from the D/A interface. On the other hand, a certain degree of mixing of the donor and acceptor phases is required to efficiently collect the carriers.

**Fig. 9:**
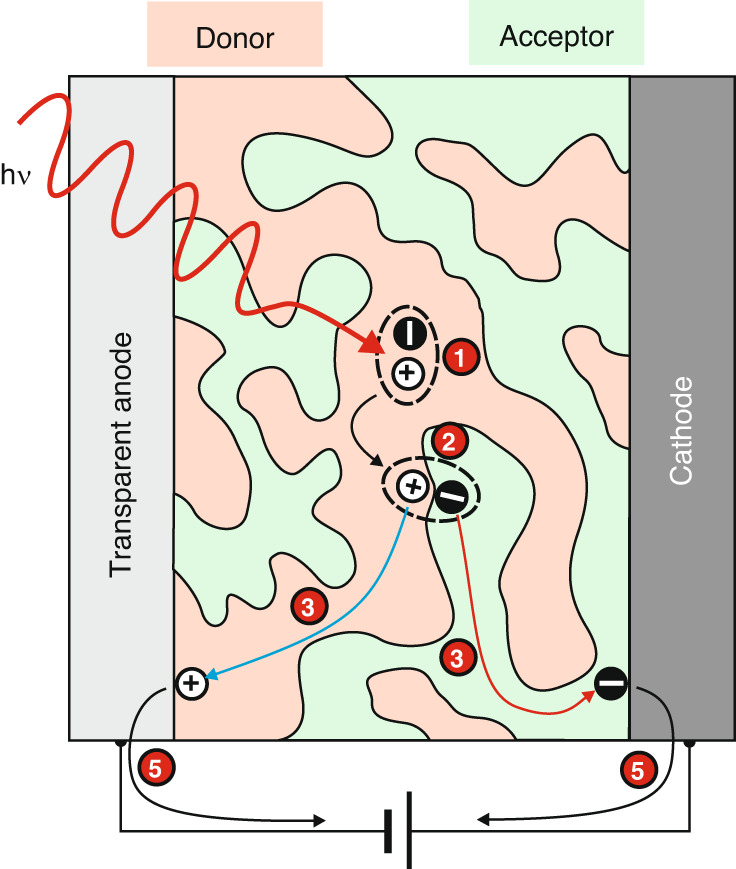
Operating mechanisms for general type o OSC photodetector. The basic processes occurring in the detector are explained in the description of Fig. 8

Figure 9 illustrates the fundamental operating mechanism of an OSC photodetector built of the photo-active layer inserted between the anode and cathode^28^. The active region possesses an interpenetrating network of acceptors and donors. The donor and acceptor domain sizes must be well monitored to improve the diffusion of excitons into the D/A interface before recombination. Appropriate interfacial layers are needed to create a low contact resistance between the absorber and electrodes for effective charge accumulation and blocking the transport of counter-type carriers to suppress dark current.

The carriers drift velocity (*v*_*d*_) is related to the electric field (*E*) by equation *v*_*d*_ = *μE*, where *μ* is the charge carrier mobility (the proportionality constant). In turn, the drift current induced by the flow of charge in an electric field depends on the mobility and carriers’ density (*n*), generated by the separation of photoexcited excitons: *J*_*drift*_ = *eμnE*. The carrier concentration results of the balance between the extraction of free carriers and the recombination of e/h pairs dissociated from excitons.

## Ultimate detectivity boundaries

**Fig. 10:**
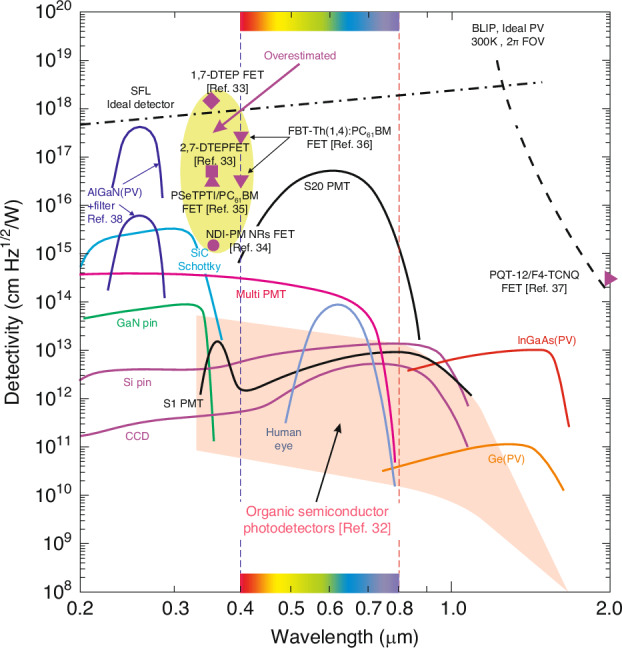
The room temperature D^*^ for OSC based detectors^32–37^ compared with typical devices (Si, AlGaN, Ge, InGaAs PDs and PMTs) for *λ* = 0.2–2 μm. The utmost BLIP and SFL are also presented. PD-photodiode, PMT-photomultiplier tube, FET-field effect transistor, PV-photovoltaic detector. The OSC photodetectors’ D^*^ marked in magenta are overestimated

The photodetectors reach the utmost performance when the device’s internal noise is lower than the photon noise^29–31^. The photon noise is not related to the imperfections of the device’s design or the integrated electronics but depends on the discrete nature of electromagnetic radiation consisting of two components stemming from the surrounding background and target. Two basic boundaries of the detector’s performance, originating from the signal fluctuation limit (SFL) of the target and the background radiation - BLIP contribution (background limited infrared photodetector). The both SFL and BLIP detectivities are presented in Fig. 10 (within 0.2‒2 μm spectral range). The SFL and BLIP curves intersect at a wavelength, *λ* ~ 1.2 μm. For <1.2 μm, the utmost detectivity weakly depends on the wavelength while for >1.2 μm (where the BLIP dominates) the D^*^(*λ*) function is strong resulting from the severe rise of the scene radiation at the edge of the 300 K background spectral distribution.

Figure 10 also assesses the detectivity of OSC based detectors with selected typical photon devices dominating the global market and operating at 300 K with 2π field of view (FOV)^32–37^. The OSC based photodetectors classified by fabricating methods are reviewed in Table 4 (according to ref. ^32^) and are marked by pink area in Fig. 10. The upper D^*^ of OSC based detectors match well with the standard performance for typical photodetectors, however, the variation stays within three orders of magnitude. AlGaN photodiodes exhibit the highest D^*^ at 260 nm, but to reach the utmost D^*^ (close to the SFL limit) it is important to incorporate filters to limit the residual solar radiation contribution^38^. The utmost D^*^ > 10^15^ Jones shown in magenta for OSC UV detectors (refs. ^33–36^) is overestimated. It should be stressed that no information is given on the use of filters to measure the photodetectors’ performance.

**Table 4:** The recent OSCs state-of-the-art/figures-of-merit and progress (Reproduced with permission for ref. ^32^ © The Royal Society of Chemistry 2022)

Active layer material	Type*	Peak [nm]	Spectral range [nm]	FWHM [nm]	Detectivity [Jones]	*EQE* [%]	Responsivity [A/W]	Bias [V]	*LDR* [dB]	*f*_‒3*dB*_	Year
**Solution broadband**											
PCDTBT:PC_61_BM		528	300–800		3.2 × 10^13^	27	0.31	‒2.0	148	91.0	2016
PCDTBT:PC_71_BM		570	400–720		3.5 × 10^13^	76	0.35	‒5.0	100	50.0	2015
PCDTBT:PC_71_BM		532	300–800		1.0 × 10^13^	70	0.30	‒1.0	180	1000	2014
PBDB-T:PbS-TBAI		630	400–1000		1.1 × 10^13^	335	1.70	‒40	65	—	2018
PBDTTT-C:PC71BD		700	350–800		1.0 × 10^13^	30	0.17	‒2.0	~140	~20	2013
NT40:N2200		720	300–850		2.6 × 10^13^	56	0.33	‒0.1	97	10	2019
PDDTT:PC_61_BM		800	300–1450		2.3 × 10^13^	25	0.16	‒0.5	100	—	2009
PDPP3T:PC_71_BM		850	300–1000		1.0 × 10^13^	28	0.19	‒0.5	148	400	2015
CS-DP:PC_71_BM		850	300–1000		8.0 × 10^12^	48	0.33	0	—	—	2018
NT40:IEICO-4F		870	300–1000		7.5 × 10^13^	57	0.40	‒0.1	123	100	2021
**Solution broadband**											
C60		370	300–700		3.6 × 10^11^	40	0.12	‒6.0	180	95	2013
ClAlPcC70		730	300–800		4.1 × 10^13^	75	0.44	‒2.0	173	779	2020
Cy7-T:C60		850	600–870		1.0 × 10^12^	23	0.17	‒2.0	100	—	2015
**Solution narrowband**											
1(Pyrl):C60	NBM	481	420–550	76	2.0 × 10^11^	18	0.07	0	—	—	2019
PCDTBT:PC_71_BM	CCN	670	610–800	85	1.8 × 10^12^	35	0.10	‒1.0	160	95	2015
DPP-DTT:PC_71_BM	CCN	950	900–1020	80	4.8 × 10^12^	7.3	0.06	‒1.0	—	—	2015
P3HT:PC_61_BM:CdTe	CCN	660	650–850	100	4.8 × 10^12^	200	1.06	‒6	220	900	2016
P3HT:PC_71_BM	CIN	650	640–700	28	1.4 × 10^11^	53500	278	‒60	160	—	2017
P3HT:PC_71_BM	CIN	800	790–800	30	5.5 × 10^11^	20000	0.85	‒50	170	—	2018
P3HT:PTB7-Th:BEH	CIN	850	830–880	27	8.0 × 10^11^	15300	105	‒13	145	—	2021
F8T2:ZnO	PM	360	350–400	20	8.8 × 10^11^	2170	6.5	‒15	—	0.1	2018
PTB7:PC_71_BM	SF	745	645–745	50	1.1 × 10^12^	4.5	0.03	0	103	39	2020
NT812:Y6	SF	860	860–960	50	1.2 × 10^13^	61	0.42	‒0.1	—	—	2020
DT-PDPP2T-TT:Y6	SF	920	860–960	40	7.4 × 10^12^	—	—	‒0.1	—	—	2020
DT-PDPP2T-TT:Y6	SF	955	860–960	50	1.6 × 10^13^	—	—	‒0.1	—	—	2020
PBDB-T:m-ITIC	SF	700	750–950	120	8.3 × 10^11^	53	0.30	0	—	80	2020
PBTTT:PC_61_BM	MC	775	700–1100	13	3.6 × 10^12^	40	0.25	0	130	—	2017
PBTTT:PC61BM	MC	960	700–1100	17	1.0 × 10^13^	24	0.19	0	130	—	2017
PDPPTDTPT:SdiCNPBI	MC	1200	1000–1700	35	1.0 × 10^10^	0.90	0.01	0	—	—	2017
PDPPTDTPT:SdiCNPBI	MC	1440	1000–1700	38	1.0 × 10^10^	0.10	1.2 × 10^‒3^	0	—	—	2017
PDPPTDTPT:SdiCNPBI	MC	1580	1000–1700	47	1.0 × 10^9^	0.05	6.4 × 10^‒4^	0	—	—	2017
PDPPTDTPT:SdiCNPBI	MC	1680	1000–1700	41	1.0 × 10^8^	0.01	1.36 × 10^‒4^	0	—	—	2017
PCDTPTSe:PC_71_BM	MC	710	650–1510	60	5.0 × 10^11^	18	0.10	0	146	—	2021
PCDTPTSe:PC_71_BM	MC	1130	650–1510		1.0 × 10^11^	6	0.05	0	—	—	2021
PCDTPTSe:PC_71_BM	MC	1360	650–1510		6.0 × 10^10^	1.9	0.02	0	—	—	2021
PCDTPTSe:PC_71_BM	MC	1510	650–1510		5.0 × 10^9^	0.15	1.8 × 10^‒3^	0	—	—	2021
**Vacuum narrowband**											
ZnPc:C60	MC	905	875–1085	43	1.0×10^11^	22	0.16	0	100		2017
TPDP:C60	MC	—	810–1550	—	—	—	—	0	—	—	2017
DCV5T-Me:C60	MC	645	400–700	84	1.9 × 10^12^	50	0.27	0	130	5000	2019
D8:C60	MC	1150	810–1150	50	4.0 × 10^10^	0.25	2.32×10^‒3^	0	—	—	2019
D8:C60	MC	1665	810–1665	50	3.0 × 10^8^	4.5×10^‒3^	6.04×10^‒4^	0	—	—	2019
ZnPc:C60	MC	877	790–1180	37	1.0 × 10^11^	10	0.07	0	120	—	2020
D6:C60	MC	1115	1020–1435	58	1.0 × 10^10^	0.15	1.4×10^‒3^	0	139	—	2020
ZnPc:C60	MC	826	826–879	17	5.0 × 10^11^	75	0.50	‒10	—	19.4	2021

^*^NBM-narrow bandgap materials, SF-self-filtering and MC-microcavity

There are several reports highlighting the overestimated performance (mainly 2D layered photodetectors) by imperfect characterization procedures, including^39–42^: (*i*) incorrect noise estimates, (*ii*) device’s active area and radiant power density miscalculation, (*iii*) conflicting measured sensitivity and noise bandwidth (BW), and (*iv*) poor photodetector’s linear dynamic range - LDR (despite a large absorption coefficient - unsuitable to intense light due to the small volume of the active region). The proper characterization methods coherent with procedures for standard bulk based detectors are mandatory. Possibly the primary cause for the D^*^ overestimates reported in refs. ^33−37^ (record > 10^18^ Jones^33^) is the lack of the photogating (*g*) effect incorporation during the shot noise and the generation-recombination (g-r) noise measurements. Ussing wrong (*I*_*sh*_ = √2*qI*∆*f*) expression for the shot noise (proper *I*_*sh*_ = √2*qgI*∆*f*) results in the fake increase of the signal to noise ratio (SNR) by √*g*. Analogous relation is observed for g-r noise (frequency, *ω* dependent) according to the formula *I*_*gr*_ = √4*qI*_*d*_*g*∆*f* /(1 + *ω*^2^*τ*^2^) .

## Operating mechanisms of OSC photodetectors

**Fig. 11:**
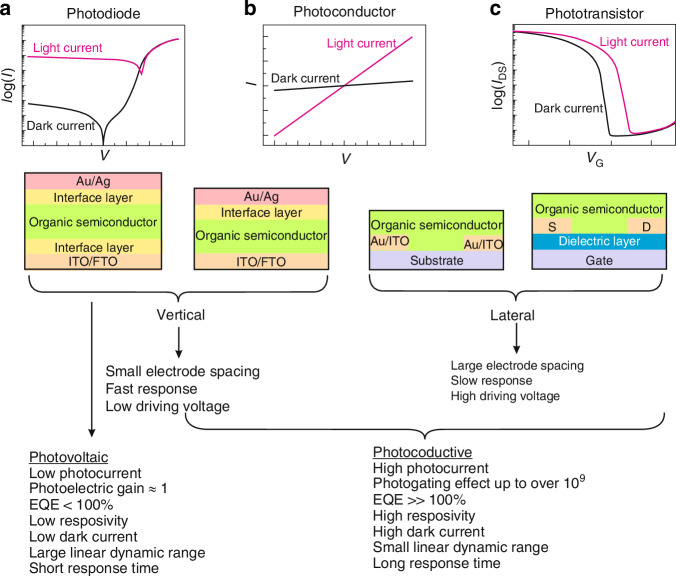
Pros and cons of the PC, PV, and PT detectors

Figure 11 shows the unique properties of OSC-based detectors. Depending on the active area and the contacts spatial arrangement, the OSC detectors may be split into lateral and vertical designs. Generally, vertical detectors require a low bias due to both short distances between electrodes and carrier transit length. Lateral photodetectors exhibit slow response and require high drive voltage related to the distance between electrodes.

The photoconductive (PC), photovoltaic (PV) and field effect transistor (FET) are the principal OSC based devices modes of operation. The PC detectors are two-terminal (two metal-semiconductor contacts) devices that allow photocurrent amplification. The PC effect results from the generation of additional free carriers (absorption of light) in the semiconductor. The needed bias to compensate for photocurrent losses (capture of carriers in defects followed by non-radiative recombination) can be high for long distance between electrodes in a planar configuration (from tens of microns to mm). Vertical photoconductors are characterized by much smaller electrode spacing (<0.5 μm).

The PV device structure, as presented in Fig. 11a, is built of absorber layer, inserted between cathode and anode. These regions are responsible for photons’ absorption, carriers’ generation, transport and extraction. PDs are characterized by low dark current and high detectivity, however, PDs suffer from low responsivity and *EQE* < 100% due to *g* ~ 1. In inorganic avalanche photodiodes (APDs) or organic photodiodes (OPDs) with photomultiplication effect, internal amplification may occur (see “Photodiodes”). Both photoconductors and FET phototransistors (PTs) exhibit high current responsivity, *EQE* (well above 100%) and high gain, however, high gain usually results in a low response time, since both gain and response time are influenced by the carrier lifetime. There are always inherent tradeoffs between response speed and responsivity. The characteristic parameters of PC and PV detectors are summarized in Table 5.

**Table 5:** The comparison of PC and PV detectors

Parametr	PC device	PV device	Schematic figures
Gain (*g*)	$g=\frac{\tau }{{\tau }_{t}}=\frac{\tau {\mu }_{e}{V}_{b}}{{l}^{2}}$^**(1)**^	$g=1$ (for APD>>1)^**(2)**^	
Responsivity (*R*)	${R}_{v}=\frac{{V}_{s}}{{P}_{\lambda }}=\frac{\eta }{{lwt}}\frac{\lambda \tau }{{hc}}\frac{{V}_{b}}{{n}_{o}}$^**(3)**^	${R}_{i}=\frac{{I}_{s}}{{P}_{\lambda }}=\frac{q{\eta }{\lambda }}{{hc}}$^**(4)**^	
Noise	$\bar{{i}_{{gr}}^{2}}=\frac{4{qIg}\Delta f}{1+{\omega }^{2}{\tau }^{2}}$^**(5)**^	$\bar{{i}_{{sh}}^{2}}=2{qI}\Delta f=2q\left({I}_{0}{e}^{{qV}/{kT}}+{I}_{0}\right)\Delta f$^**(6)**^	
Noise equivalent power (*NEP*)	${NEP}=\frac{{v}_{n}}{{R}_{v}}$^**(7)**^	${NEP}=\frac{{i}_{n}}{{R}_{i}}$^**(8)**^	
Detectivity (D^*^)	${D}^{* }=\frac{{\left(A\Delta f\right)}^{1/2}}{{NEP}}=\frac{{R}_{v}{\left({lw}\Delta f\right)}^{1/2}}{{\left(\bar{{v}_{J}^{2}}+\bar{{v}_{{gr}}^{2}}\right)}^{1/2}}$^**(9)**^	${D}^{* }=\frac{{\left(A\Delta f\right)}^{1/2}}{{NEP}}=\frac{\eta \lambda q}{{hc}}{\left(\frac{4{kT}}{{R}_{0}A}+2{q}^{2}\eta {\phi }_{b}\right)}^{-1/2}$^**(10)**^	
BLIP detectivity	${D}_{{BLIP}}^{* }=\frac{\lambda }{2{hc}}{\left(\frac{\eta }{{\phi }_{B}}\right)}^{1/2}$	${D}_{{BLIP}}^{* }=\frac{\lambda }{{hc}}{\left(\frac{\eta }{{2\phi }_{B}}\right)}^{1/2}$	
SFL detectivity	${D}_{{SFL}}^{* }=\frac{\eta \lambda }{{2}^{3/2}{hc}}{\left(\frac{A}{\Delta f}\right)}^{1/2}$	${D}_{{SFL}}^{* }=\frac{\eta \lambda }{{2}^{3/2}{hc}}{\left(\frac{A}{\Delta f}\right)}^{1/2}$	

^(1)^*τ* carrier lifetime, *τ*_*t*_ carrier transit time, *l* carrier transit length, *μ*_*e*_ carrier mobility, *V*_*b*_ voltage^(2)^, APD avalanche photodetector^(3)^, *V*_*s*_ the output rms voltage, *P*_*λ*_ incident irradiance power, *η* quantum efficiency (QW), *w* detector width, *t* detector thickness, *λ* incident light wavelength, *h* Planck constant, *c* speed of light, *n*_0_ majority carrier concentration in the n-type material^(4)^, *I*_*s*_ output rms current^(5)^, *q* electron charge, $\bar{i}$ average current, *g* PC gain, *Δf* detector’s operating bandwidth^(6)^, $\bar{{i}_{{sh}}}$ shot noise, *I* total current, *V* voltage, *k* Boltzmann constant, *T* operating temperature^(7)^, *v*_*n*_ rms noise voltage, *R*_*v*_ voltage responsivity^(8)^, *i*_*n*_ rms noise current, *R*_*i*_ current responsivity^(9)^, *A* detector’s photosensitive area, $\bar{{v}_{J}}$ Johnson noise voltage, $\bar{{v}_{{gr}}}$ generation-recombination noise voltage^(10)^, *R*_0_*A* product of zero-bias resistance and photosensitive area, *ϕ*_*b*_ the background radiation flux density

**Fig. 12:**
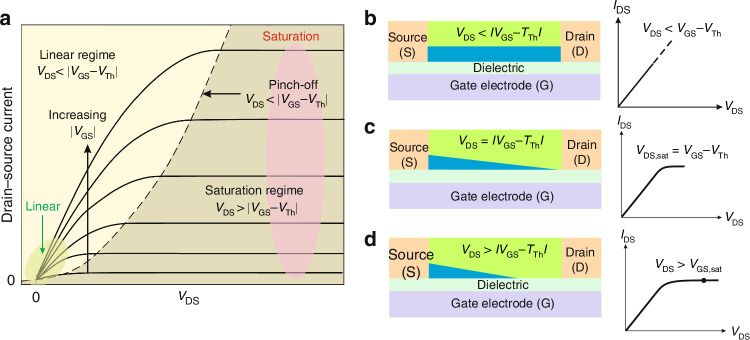
Operation modes of OFET device. **a** Ideal output source-drain current characteristics for different gate voltages. **b** The linear mode. **c** At pinch-off. **d** The saturation mode

Another structure is the organic field-effect-transistor (OFET). The structure of OFET comprises source, drain, gate, insulation layer, and semiconductor layer. This transistor configuration aids in suppressing dark current while enhancing photocurrent. The crucial difference with FET-based devices is that OFET operates in the accumulation mode. FET device operates under two voltages: the drain-source voltage (*V*_*DS*_) and the gate-source voltage (*V*_*GS*_). If *V*_*GS*_ = 0 V the transistor is “off ” (flat-band condition requires threshold voltage *V*_*th*_ to fill the charge traps at the semiconductor-dielectric interface). The applied positive (negative for a p-channel transistor) *V*_*GS*_ will accumulate charge at the semiconductor-dielectric interface (transistor is “on”) and *V*_*DS*_ will proportionally define the drain-source current, as presented in Fig. 12a, b. Both the majority carrier concentration in the transistor channel and the drain-source current will be influenced by the electric field generated by *V*_*GS*_. The threshold voltage (*V*_*th*_) is the minimum *V*_*GS*_ that is needed to establish a conducting path between the source and drain. When the *V*_*DS*_ reaches |*V*_*GS*_ – *V*_*th*_ | , the region near the drain electrode is pinched-off [Fig. 12c], while the drain-source current saturation is visible [Fig. 10a] versus *V*_*DS*_ [Fig. 12d].

**Fig. 13:**
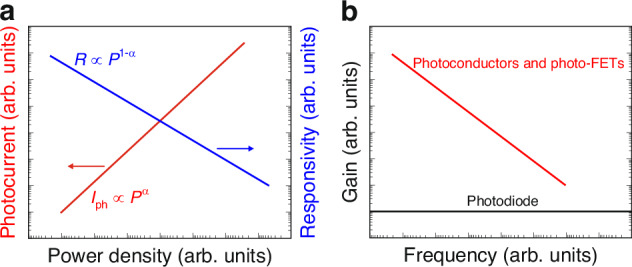
The performance of the photodiodes, photoconductors and photo-FETs. **a** Photocurrent/responsivity versus radiation power. **b** Gain versus frequency

FET designs allow to improve responsivity. Those detectors exhibit a limited LDR caused by the charge relaxation time (available photoexcitation states are saturated causing responsivity decrease versus optical power). The high-performance devices exhibiting a wide LDR [the photocurrent exhibits a linear dependence on the incident radiation power before absorption saturation (*I*_*ph*_ ∝ *P*^*α*^, where *α* is close to 1)] are required for demanding applications. In terms of the OSC photodetectors a complex carrier generation-recombination and trapping mechanisms drives exponent 0 < *α* < 1 driving the detector’s responsivity according to the relation *R* = *I*_*ph*_/*P*, so *R* ∝ *P*^*−(*1*−α)*^. Figure 13a presents the net photocurrent and responsivity nonlinear dependence versus light power. Similar dependence is visible for photoelectric gain being greatly contributed by traps [see Fig. 13b]. If light power increases, the carriers are gradually filling traps decreasing photoelectric gain and carrier lifetime. The sensitivity is not influenced by low radiation powers (due to the high trap states density). Normally, the sensitivity estimated for different light powers is not used for detectors performance comparison. The current responsivity is estimated usually by the power densities several orders of magnitude lower than 1 mW/cm^2^ ^43^.

## Design and OSC detectors performance

There are published review papers on the OSCs progress covering selected topics to include material physics, *processing* device designs and possible applications^6–8,10,32,44,45^. Therefore, the aim of this paragraph is to organize this data by presenting the basic structures of organic photodetectors, reviewing their latest performance and identifying both promises and challenges. Particular attention was paid to examples of overestimation of the performance of photodetectors, mainly FET phototransistors.

**Fig. 14:**
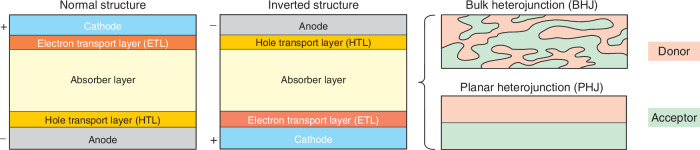
Schematic structures of OPDs: the standard and inverted structures with the BHJ and PHJ for the active/absorption layer (Reproduced with permission for ref. ^44^ © 2022 Shan, Hou, Yin, Guo)

Figure 14 shows schematic device structures of OSCs photodiodes. As shown, these structures contain active layer inserted between two contact regions (cathode and anode) with electron transport layer (ETL) and hole transport layer (HTL) to guarantee effective carrier extraction. ETL facilitates the movement of electrons while effectively blocking the transport of holes. Typically, this layer necessitates high transmittance, high electron mobility, suitable energy level alignment, as well as robust photochemical and thermal stability. Depending on the cathode/anode positions, both the standard design (top cathode/bottom anode) and an inverted one (bottom cathode/top anode) are processed. Both bulk (B) and planar (P) heterojunction (HJ) consisting of donors and acceptors (polymers and small molecules), were implemented as active layers. Compared to the PHJ, the BHJ has an interpenetrating network of donors and acceptors, providing more efficient D/A coupling, collecting more excitons and generating more photogenerated e/h pairs.

**Fig. 15:**
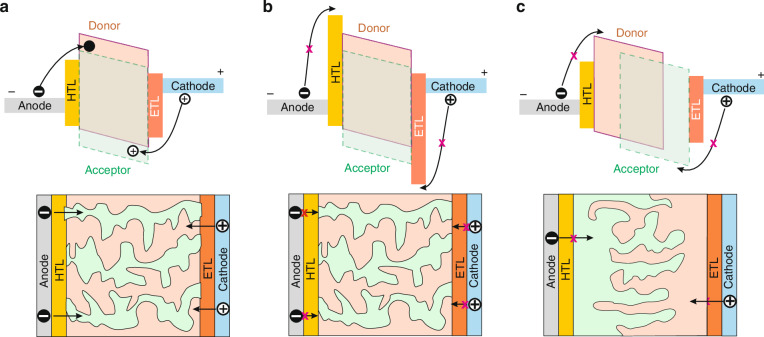
The carrier injection mechanism in OSCs photodiodes. **a** BHJ with percolating networks. **b** BHJ with blocking layers. **c** Quasi-planar heterojunction (q-PHJ) with vertical phase segregation (Reproduced with permission for ref.^44^ © 2022 Shan, Hou, Yin, Guo)

To suppress the dark current of OSCs photodetectors, several techniques were introduced to minimize the trap density, optimize the absorber phase morphology and select phases for efficient carrier transport^45,46^. In a well-chosen and mixed BHJ, the dark current under negative voltage can originate from the unwanted carrier injection from the contact. This process is presented in Fig. 15a: the injection of electrons from the anode to LUMO and holes from the cathode to the HOMO. One way to limit the dark current (to reduce unwanted carrier injection paths) is the carriers’ injection blocking at the electrodes [Fig. 15b]. Increasing the energy barrier of the HTL may limit undesired carrier injection and facilitate the photogenerated carriers’ extraction (HTL serves as an electron blocking layer—EBL). For this reason, a particular challenge is the selection of appropriate interfacial layers with suitable carrier mobility and energy levels.

Another way to eliminate unwanted carrier injection pathways is a q-PHJ with phase segregation control, as presented in Fig. 15c. In this case, pure D/A phases are appropriately placed in areas near the anode/cathode, while the central region encompasses the mixed D/A interfaces to intensify photocurrent generation. The development of q-PHJs requires the subsequent deposition of polymeric donor and a small-molecule acceptor along with appropriate morphological engineering.

### Photoconductors

Photoconductor consists of simple semiconductor active layer with two electrodes (ohmic contacts). The contact ohmicity provides electrical neutrality meaning that for every electron transported to the electrode, another one will be injected at the second contact until e/h recombination occurs. Photoconductors are intended to trap minority carriers causing the majority charge carriers to recirculate through the device until recombination meaning that the photogenerated carrier may be collected multiple times. Consequently, a photoconductive gain *g*, is observed, which can be simply described by the ratio of recombination time (*τ*) to the transit time (*τ*_*t*_); *g* = *τ*/*τ*_*t*_^47^. Unlike photodiodes, for which typically *g* ≈ 1, photoconductive detectors can exhibit *g* > 1 and *EQE* > 100%. As is shown in Fig. 13, more trapping sites are occupied versus light power and gain gets lower, but the device gets faster. Thus, a trade-off exists between *g* and BW.

**Fig. 16:**
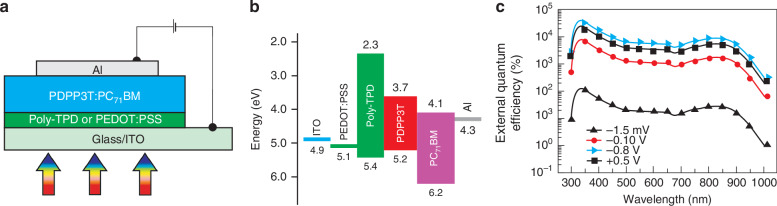
The structure of OSCs based photodetector ITO/ZnO/PDPP3T:PC71BM/A with a broad response and high gain. **a** Device structure of the broadband photodetector. **b** Energy diagram for constituent materials. **c** EQE spectra measured under selected voltages for UV (Reproduced with permission for ref.^50^ © 2016 WILEY-VCH Verlag GmbH & Co. KGaA, Weinheim)

Variation in carrier mobility is easily met in OSCs by introducing a mixture of materials in the active region of detector. The ratio D/A can be tuned accordingly to minimize the formation of percolation paths for one of the carriers. Using this procedure, *EQE* ~ 37,500% was reached by BHJ 100:1 with P3HT and PC_61_BM^48^. One of the highest photoconductive gain ~10^5^ was obtained using a BHJ-based lateral detector configuration with poly dithienobenzodithiophene-co-diketopyrrolopyrrolebithiophene (PDPDBD) and PC_71_BM^49^. That remarkable value was reached for low light power ~20 nW/cm^2^. Figure 16 shows, the structure of OSCs based photodetector ITO/ZnO/PDPP3T:PC_71_BM/A with a broad response and high gain. The device showed a large *EQE* ~ 140,000%^50^.

### Photodiodes

Most of the OSCs based photodiodes reach *EQE* < 100%. The avalanche multiplication is not used in OSCs (excitons high binding energy) to improve device’s sensitivity. The photodiodes are characterized by low power dissipation, insignificant 1/*f* noise, inherent high impedance, simple multiplexing via readout integrated circuits (ROICs), and are typically used for large arrays. The reverse-biased photodiodes exhibit high impedance allowing better electrical matching with compact/low-noise silicon readout preamplifier circuits. The photoresponse is linear for much higher photon flux than for photoconductors, due to higher doping levels (active layer) and fast photogenerated carriers’ collection by the electric field dropping on the junction.

The response speed of OSCs photodiodes is limited by the *RC* time of the measurement set-up (contact resistance, device’s sheet resistance and load resistance) and the carriers’ transit time (or combined). The carriers’ transit time is conditioned by the charges’ mobility. The thicker device the longer transit time being undesirable and that trade-offs are required as for those related charge collection.

Table 4 primarily collects the performance of OSCs’ photodiodes fabricated by selected methods. Photoconductors reach *EQE* > 100% where photoconductive gain is observed.

**Fig. 17:**
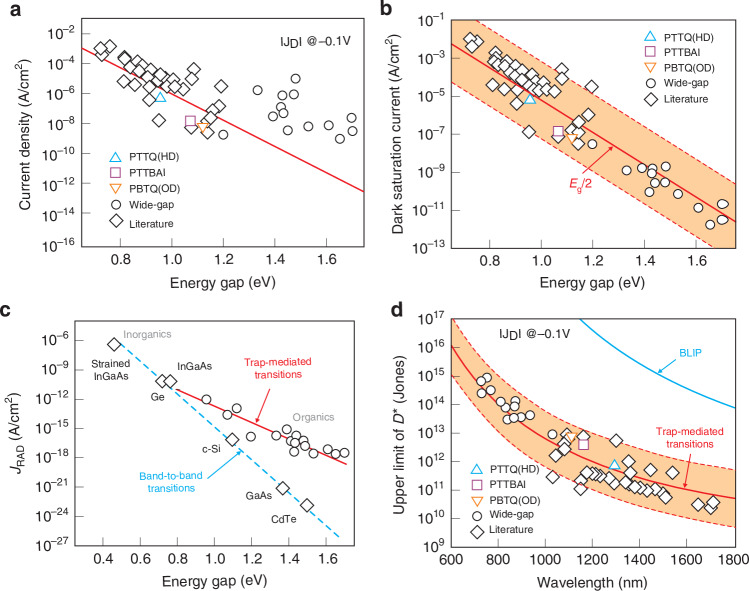
The mid-gap trap states contribution to the OSC based photodiodes performance. **a** Dark current densities curves (square, triangles and circles based on ref. ^51^; diamonds from the selected published papers). **b** Extracted dark saturation current density versus energy gap (the red solid line corresponds to a trend line ${J}_{d}={J}_{0}\left[\exp \left({qV}/2{kT}\right)\right]$ with factor *J*_*0*_ = 2 × 10^3^ A/cm^2^, a shaded area corresponds an upper level *J*_*0*_ = 2 × 10^5^ A/cm^2^ and lower level *J*_*d*_ = 20 A/cm^2^). **c** The trends for band-to-band (activation energy *E*_*a*_ = *E*_*g*_) and mid-gap transitions (*E*_*a*_ = *E*_*g*_ /2) are shown by the blue dashed and red solid lines. **d** The estimated upper limit of detectivity − simulated based on the red solid line in Fig. b, the red shaded region corresponds the shaded area in Fig. b (Reproduced with permission for ref. ^51^ © 2023 Sandberg, Kaiser, Zeiske, Zarrabi, Gielen, Wouter Maes, Vandewal, Meredith, Armin)

The high performance requires low dark current. In comparison with ISCs typical photodiodes, the OSCs devices exhibit larger dark currents than stemming from thermal radiative transitions. Sandberg et al. presented (analysis of the temperature-dependent dark current curves of the narrow gap OSC photodiodes) that the thermal activation of the dark current for low reverse voltage results from mid-gap states transitions^51^. Figure 17a shows dark current densities for selected OSC based photodiodes (triangles, square and circles are from ref. ^51^; diamonds − from the selected papers).

Assuming that the photodiodes performance is primarily driven by mid-gap trap states, the dark current may be given according to the relation:

$${J}_{d}={J}_{0}\left[\exp \frac{{qV}}{{mkT}}-1\right]$$

where: *J*_0_ corresponds to the dark current saturation density and *m* is diode ideality factor (for mid-gap states *m* = 2). As shown in Fig. 17, for low forward and reverse biases, the proper agreement is reached between experimental data and the analytical model with *m* = 2. Large deviation between analytical and experimental data is observed at high reverse bias probably due to shunt resistance influence. Also, the transitions between the states of the mid-gap set an upper detection limit [Fig. 17d], which is, however, well below the BLIP limit.

The spectral response characteristic of OSC photodiodes ranges from 200 to 1500 nm and their spectral characteristics can be tuned by the active layers’ material selection. The selection/design of absorber allows to develop broadband and selective photodetectors exhibiting flexibility, high thermal stability, fast response and high sensitivity^10,32^.

The broadband OSCs photodiodes have attracted attention due to wide applications in image sensors, health monitoring, machine vision and night surveillance. Compared to narrowband devices, it is much simpler to fabricate broadband OSC photodiode by absorber materials exhibiting wide response range.

Many OPD structures were reported—see refs. ^10,32,44,52,53^. Most of them are adapted from technological advances of the solar cells to include perovskites.

**Fig. 18:**
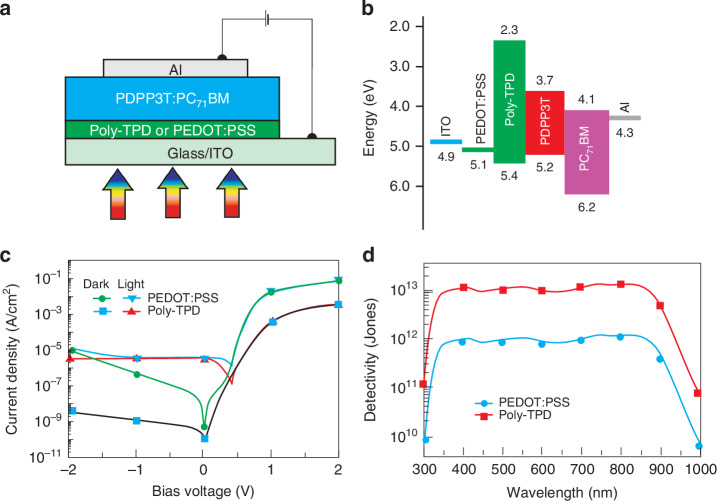
Photodiodes with the low-bandgap PDPP3T polymer blended with PC_71_BM. **a** Device structure layout. **b** Energy band levels diagram for OSCs photodiode fabrication. **c** *J-V* characteristics measured in dark and under 850 nm light conditions (2.7 μW). **d** Calculated detectivity for poly-TPD or PEDOT:PSS as the anode interlayer under −0.5 V bias (Reproduced with permission for ref. ^54^ © Wiley)

Zhou et al. developed OSC based photodiodes with broadband spectral response covering from 300 to 1000 nm. Photodiodes with low-bandgap PDPP3T polymer blended with PC_71_BM are shown in Fig. 18^54^. In this structure, thin poly[N,N′-bis(4-butylphenyl)N,N′-bis(phenyl)-benzidine] (poly-TPD) or PEDOT:PSS is used as the EBL. It appears, that the dark current of the OPD with poly-TPD as EBL is 2860 times lower than that devices with PEDOT:PSS as EBL. This result indicates the superb EBL capability of the poly-TPD what is shown in Fig. 18c, however the decrease of dark current is explained by increase of shunt resistance for the OPD with poly-TPD as EBL. The suppression of dark current and well-preserved light current are beneficial for improving the detectivity. The calculated detectivity of OPDs reaches ~10^13^ Jones for wavelengths 350–870 nm using poly-TPD as EBL and is over more than one order of magnitude higher than that for devices with PEDOT:PSS as EBL [see Fig. 18d].

**Fig. 19:**
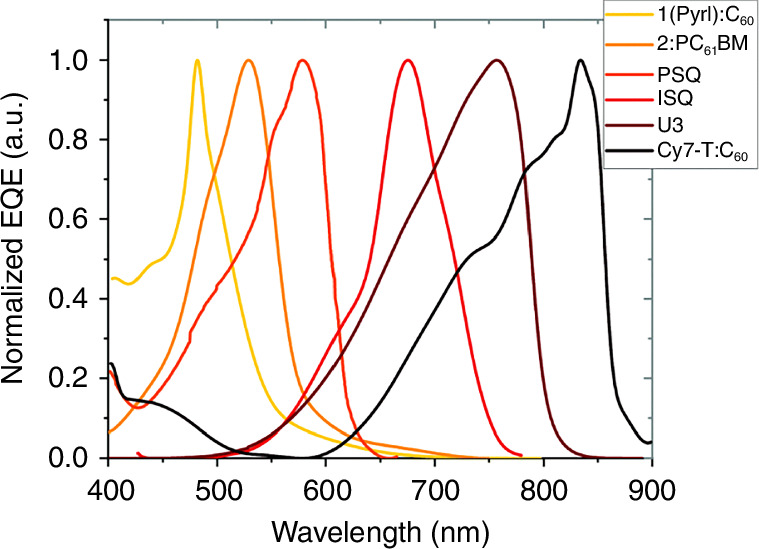
Normalized *EQE* for OSCs narrow-photo-absorbing based photodiodes versus wavelength. 1(Pyrl):C_60_ and Cy7-T:C_60_ are PHJs (2:PC_61_BM is a BHJ blend). PSQ and ISQ are single-component photo-absorbing materials with D/A/D chemical structure and U3 is also single component device (Reproduced with permission for ref. ^32^ © The Royal Society of Chemistry 2022)

Narrowband detection capability is being intensively developed for many applications to include the most important—colour imaging. Also, OSCs can be used to achieve narrowband sensitivity by tuning their molecular electronic structure. In this respect, single-component systems have presented potential advantages. Figure 19 presents normalized *EQE* of typical narrowband absorbing compounds, however, the narrowband absorbing suffers from lack of active layer materials operating for wavelengths >1000 nm.

In general, it is difficult to find donor and acceptor pairs that match both in absorption range and energy levels for the needed performance. From these reasons, to circumvent the material limitation, other engineering methods have been chosen to realize colour discrimination. The most important are:

- Charge injection narrowing (CIN),
- Charge collection narrowing (CCN),
- Microcavity enhancement,
- Self-filtering,

being systematically summarized in ref. ^32^ addressing all features from device structure engineering and implemented materials.

**Fig. 20:**
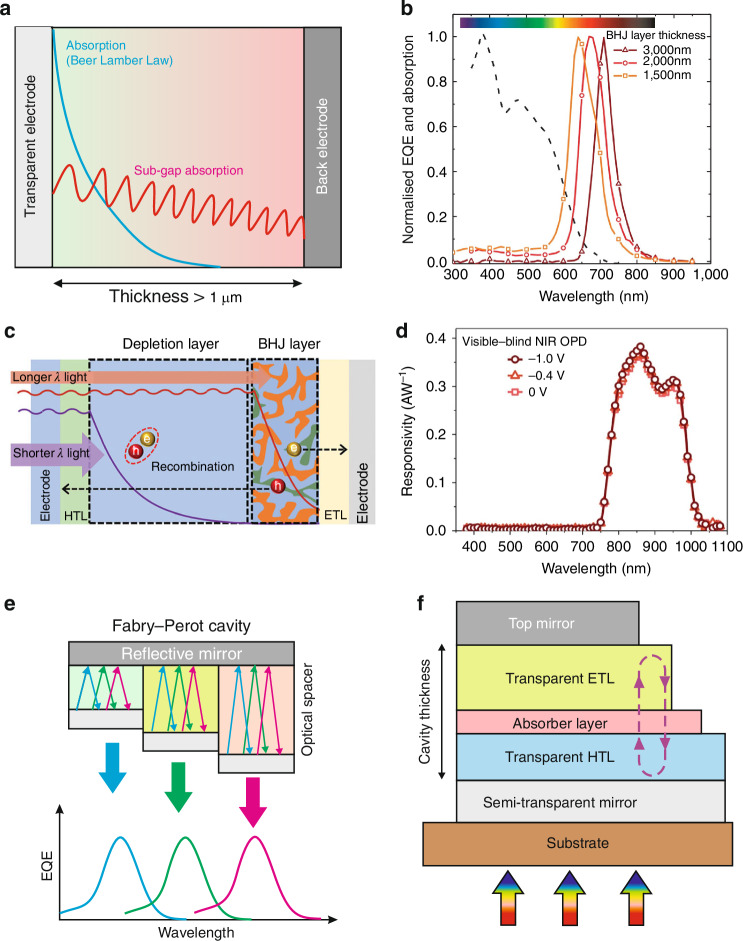
Operation principle of narrow-band OPDs structures. **a** The schematic structure of CCN photodiode. **b** Normalized *EQE* for selected absorber thicknesses (Reproduced with permission for ref. ^55^ © Nature). **c** Self-filtering photodiode operating mechanism. **d** Responsivity spectra measured for the filter-free visible-blind NIR OPD operated under selected voltages: 0, -0.4 and -1 V (Reproduced with permission for ref. ^56^ © Wiley). **e** Fabry-Perot cavity (the length of optical spacer determines the resonance wavelength) operating fundamentals. **f** Fabry-Perot cavity in a photodetector with partially transparent bottom mirror (electrode)

Figure 20 shows the operation principle of selected narrow-band OPDs^55,56^. As presented in Fig. 20a, CCN involves manipulating the efficient accumulation of charges in the active layer. The shorter wavelength photons are absorbed near the front transparent contact, while sub-gap energy photons penetrate the entire absorber reaching the reflecting back contact and the photogenerated carriers are effectively extracted creating a peak photoresponsivity located near the absorption edge of the absorber. By changing the active layers thickness, the extraction of the photogenerated carriers in the active area is altered, resulting in a shift of the photoresponse peaks [Fig. 20b].

The conventional CCN strategy requires a thicker absorber (above 1 µm), what limits the device’s responsivity and response time (thin active layer architecture is beneficial to improve the photoresponse performance). Figure 20c presents narrowband self-filtering OSC photodiode with a 430-nm thick P3HT depletion layer linked with a 120 nm thick PBDB-T:m-ITIC BHJ. As is shown in Fig. 20d, that absorber heterostructure was found to absorb light for wavelengths <600 nm and a marked increase in photoresponse appeared in the deep red region.

In the case of the CCN method, spectral tunability is relatively limited to a small bias voltage, where sensitivity is relatively low, making it inacceptable for applications. Optical resonant cavity (ORC) or optical tuning microcavity were found to be more versatile technique for improving spectral selectivity. Two mirrored metal electrodes (one of which is semi-transparent) building a Fabry-Perot cavity is presented in Fig. 20e. The device structure is presented in Fig. 20f. The cavity length and the refractive index of spacer located between the two electrodes drives the resonant wavelength enhancing significantly the optical transmission.

**Fig. 21:**
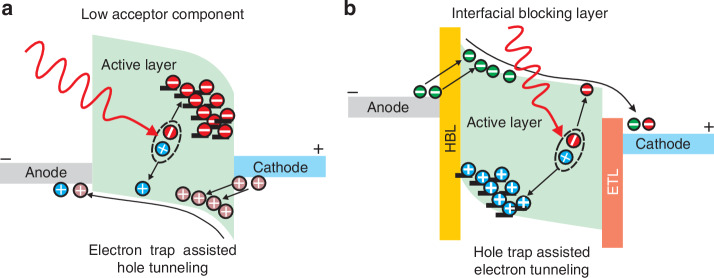
Energy band profiles and operating mechanisms for photomultiplication effect in OPDs. **a** Ultra-low concentration of one component (example of acceptor). **b** Interfacial blocking layer (HBL) (Reproduced with permission for ref. ^44^ © 2022 Shan, Hou, Yin, Guo, corrected publication 2022)

Generally, photodiodes exhibit modest photoresponse and zero voltage operating condition. To increase sensitivity/responsivity, photomultiplication is required to allow internal photocurrent amplification (as used in ISC photodiodes). Unlike the ISCs counterparts, avalanche effect is problematic in OSCs due to the large exciton binding energy. It is possible to achieve photomultiplication (PhM) by trap-assisted tunnelling. Figure 21 presents two mechanisms where isolated ultra-low concentrations donor or acceptor islands are created preventing the dissociated carriers from moving to the electrode being related to the discontinued transport pathways. Acceptors create the charge trapping states at the active layer/cathode interface, and the accumulated electrons introduce localized field and bending the interfacial energy level making the hole tunnelling possible by reverse voltage. The second mechanism is shown in Fig. 21b, where hole buildup at the active layer and the hole blocking layer (HBL) interface. Electron tunnelling injection may also occur. The net current is generated by photogenerated carriers and injected by tunnelling allowing to achieve photocurrent gain >1^57^. The disadvantages of these photomultiplication are usually large dark current and long response time hindering potential applications^44^.

**Fig. 22:**
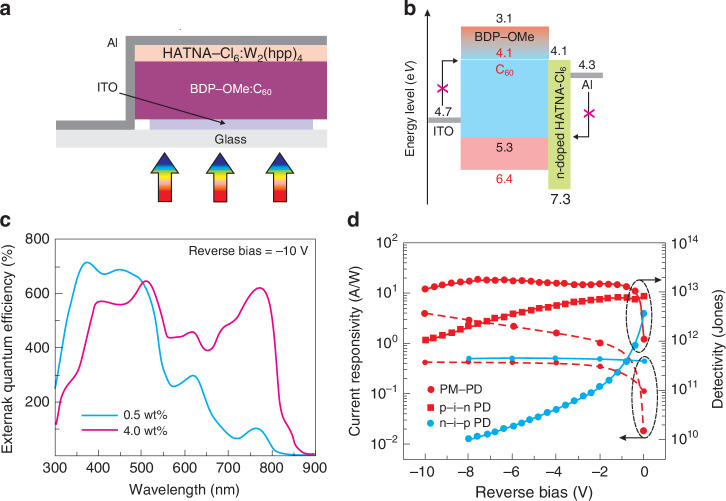
Photomultiplication-type BDP-OMe:C_60_ photodetector. **a** Device structure. **b** Device band energy diagram. **c** *EQE* for selected donor concentration under − 10 V. **d** Comparison of current responsivity and detectivity versus reverse voltage for devices: PM-PD, p-i-n PD and n-i-p PD (Reproduced with permission for ref. ^58^ © 2022 Xing, Kublitski, Hänisch, Winkler, Li, Kleemann, Benduhn, Leo. Advanced Science published by Wiley-VCH GmbH)

An example pf photomultiplication-type OSCs photodetector based on the BDP-OMe:C_60_ system is shown in Fig. 22 (BDP-OMe donor, C_60_ acceptor). The low level of the BDP-OMe content is used to substantially reduce the percolation paths and introduce hole traps. Figures 22a, b show a layer sequence consisting of ITO/BDP-OMe:C_60_ (4.0:96.0 wt%, 400 nm)/HATNA-Cl_6_:W_2_(hpp)_4_/Al. The last layer (HATNA-Cl6:W_2_(hpp)4/Al) is the ETL and HBL between the active layer and the Al electrode to enable electron transport and limit the reverse dark current [dark current is low due to the high barrier (>0.6 eV) to charge injection in reverse voltage]. The lack of a percolation network for photogenerated holes (under light condition) results in a strong hole-trapping effect shifting the trapped holes the HOMO of C_60_ upward close to the anode. At a low reverse bias, the electron injection barrier on the anode side becomes thin enough allowing electrons to tunnel into the active layer.

The PhM-OPD spectral responsivity is within UV − NIR ranges. As is shown in Fig. 22c, a low donor content (4.0 wt%) in the BDP-OMe:C60 contributed to *EQE* beyond 100% at low bias of −1 V. For wavelengths <550 nm, strong absorption is observed for both 0.5 wt% and 4.0 wt% donor contents in active region. The device with 4.0 wt% extends absorption to the NIR region showing 600% amplification at 780 nm. In comparison with two another optimized OPDs (p-i-n and n-i-p- architectures), the PhM-OPD reaches superior performance, what shows Fig. 22d. Both current responsivity and detectivity are comparable to Si photodiodes (D^*^ > 10^13^ Jones).

### FET phototransistors

As noted in “Operating mechanisms of OSC photodetectors”, the FET phototransistor is three-terminal device (gate, source electrodes, drain) where the channel resistance (between the source and drain) can be adjusted by the gate voltage. The transport channel may be altered by the light absorption in OSC. The phototransistor exhibits high photocurrent and high responsivity, high internal photoconductive gain leading to photocurrent enhancement and *EQE* > 100%. To reach high photosensitivity the channel material should exhibit high carrier mobility and light absorption. The development has been reached via modification of the film morphology and design of molecular structures.

Organic FET phototransistors are typically thin devices and are therefore more susceptible to local electric fields than conventional bulk materials. Under these conditions, the photogating effect can strongly modulate the channel conductance by the external gate voltage. High optical gain is particularly important because quantum efficiency is limited in a thin device despite a strong absorption coefficient.

Table 6 presents the utmost performance of OSCs phototransistors. Detectivity is also marked in Fig. 10^33–37^ where some published results are overestimated - close or even surpassing the ultimate SFL and BLIP conditions [SFL^33^ and BLIP^37^]. The reasons for unrealistic detectivity are discussed in “Operating mechanisms of OSC photodetectors”. Below we will quote selected characteristics of OSC FET phototransistors.

**Table 6:** The utmost performance of OSCs based FET phototransistors

Material	Responsivity [A/W]	*g*/*EQE* [%]	Detectivity [Jones]	Response time	Wavelength [nm]		ref.
2,7-DTEP	1.04 × 10^5^	‒	5.28 × 10^16^	‒	370		^33^
1,6-DTEP	2.86 × 10^6^	‒	1.49 × 10^18^	‒	370		^33^
NDI-PM NRs	7.23 × 10^3^	2.5 × 10^6^ %	1.4 × 10^15^	≈ 250 ms	365		^34^
PSeTPTI/PC_61_BM	2.2 × 10^4^	7.5 × 10^4^	3.1 × 10^16^	≈ 3 s	365		^35^
FBT-Th4(1,4):PC61BM	1.2 × 10^5^	3.7 × 10^5^	3.18 × 10^16^	≈ 300 ms (*V*_*G*_ = 30 V)	410		^36^
FBT-Th4(1,4):PC61BM	1.6 × 10^4^	5.0 × 10^4^	3.3 × 10^17^	≈ 40 ms (*V*_*G*_ = 0 V)	410		^36^
PQT-12/F4-TCNQ	2.75 × 10^6^	10^8^ %	3.12 × 10^14^	≈ 10 ms	2000		^37^

**Fig. 23:**
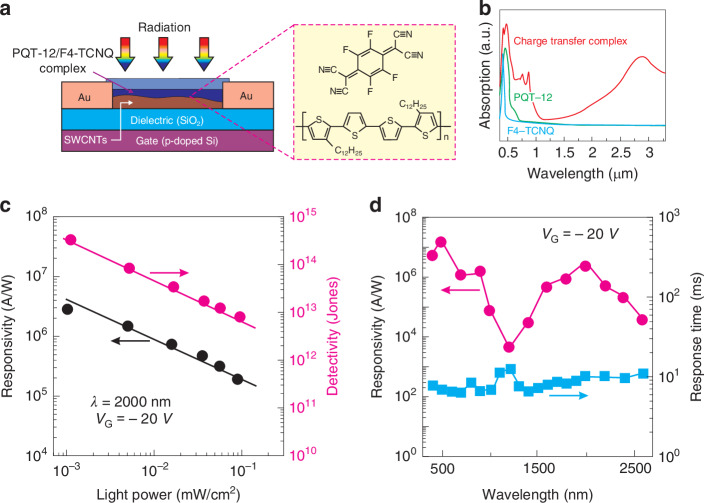
The OSCs phototransistor. **a** Design of the PQT-12/F4-TCNQ phototransistor, on right: molecules structure of F4-TCNQ and PQT-12. **b** Absorption spectra of the F4-TCNQ film, PQT-12 film, and PQT-12/F4-TCNQ film. **c** The current responsivity and detectivity versus light power for *λ* = 2000 nm and *V*_*G*_ = ‒20 V (black dots - responsivity, red dots - detectivity). **d** Current responsivity and response time versus wavelength for *V*_*G*_ = ‒20 V (Reproduced with permission for ref. ^37^ © Wiley)

Figure 23 presents the design and performance of high-quality NIR phototransistor. The active region consists of ternary semiconductor organic D/A complex and semiconducting single-walled carbon nanotubes (SWCNTs)—see Fig. 23a. The SWCNTs solution was homogeneously dispersed on the Si/SiO_2_ substrate by drop-coating method, and 40 nm thick Au contacts were thermally evaporated. As the final processing step, the PQT-12/F4-TCNQ charge transfer complex solution was spin-coated on the top surface, followed by thermal annealing. As Fig. 23b shows, the absorption spectra of PQT-12 films, F4-TCNQ films, and PQT-12/F4-TCNQ complex films combining the absorption spectrum in UV − VIS − NIR ranges. The largest photoresponse was observed for 500 nm, while the maximum response time was reached for 2000 nm. This NIR response is enhanced by the D/A charge transfer complex, whereas the SWCNTs based devices exhibited low response.

The experimental data presented in Fig. 23c, d shows a large photogating effect in OSCs phototransistors, which influences the capability to reach high current responsivity. At low light excitation intensity, the current responsivity greater than 10^6 ^A/W may be reached at *λ* = 2000 nm and *V*_*G*_ = ‒20 V, whereas for 500 − 1000 nm wavelength, the responsivity > 10^7 ^A/W [see Fig. 23d]. The responsivity improvement by photogating results in the limited dynamic range (LDR) related the charge relaxation time, what was discussed in “Operating mechanisms of OSC photodetectors” [see Fig. 13b]. In the case of the device in question, the response time reaches ~10 ms [see Fig. 23d], however, in other cases of OSCs phototransistors, the response times to several seconds was reported (see Table 6). The estimated detectivity above 10^14^ Jones for the photodetector at *λ* = 2000 nm is overestimated due to the physical BLIP limitation for 2π FOV—see Figs. 10 and 23c.

**Fig. 24:**
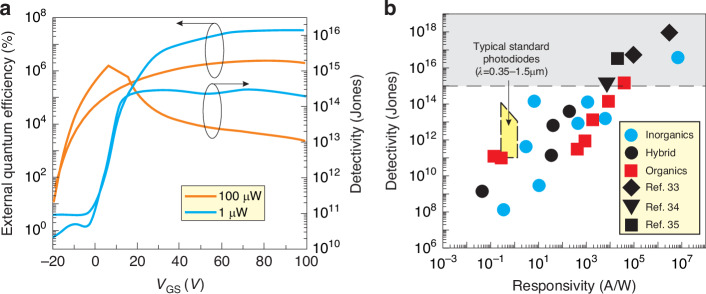
Characteristics of organic FET phototransistors. **a** *EQE* of naphthalene diimide-phenylmethyl (NDI-PM) nanorod (NR)-based phototransistor for UV light powers 100 and 1 μW cm^-2^. **b** Detectivity versus current responsivity for VIS-blind UV photodetectors presented in refs. ^33–35^ The measured results within the grey box are overestimated

To verify the photoconductor/phototransistor’s performance, the *EQE* may be assessed as important benchmark. Since the current responsivity is given by the formula:

$$R=\frac{q\lambda \eta }{{hc}}g=\frac{q\lambda }{{hc}}{EQE}$$

it follows that *EQE* = *ηg*, where *η* is the internal quantum efficiency (*IQE*). Increase in quantum efficiency > 100% (when *g* ~ 1) is related to the photoconductive gain enhancement (FET phototransistors - photogating effect) reaching *g* ~ 10^6^ at low excitation levels [see Fig. 24b]. It is believed that the large photoconductive gain in FET phototransistors not assumed in the shot/g-r noises estimates, is the main reason for OSCs based photodetectors detectivity overestimates depicted in Fig. 24. For comparison, Fig. 24b presents standard photodetectors (photodiodes) detectivity and current sensitivity for 350‒1500 nm. For the high *g* range (and responsivity), the FET phototransistors detectivity is significantly higher than those for typical photodetectors. The measured results marked in the grey box are overestimated.

## Conclusions

OSCs developed from a niche research subject as a favourable alternative to the photovoltaic and photodetector markets. OSCs performance has been significantly improved, benefiting from the rapid progress in design and synthesis of new OSCs for 40 years. They have been developed to realize detectors being able to circumvent some limitations of their ISCs counterparts. The spectral response of OSCs materials has been extended from UV to NIR ranges. OSCs may be widely used in wearable optoelectrical devices, medical diagnostics, optical imagers, spectrometers, and light communications being related by mechanical flexibility, spectral tunability, light-weight and simple fabrication/processing.

**Fig. 25:**
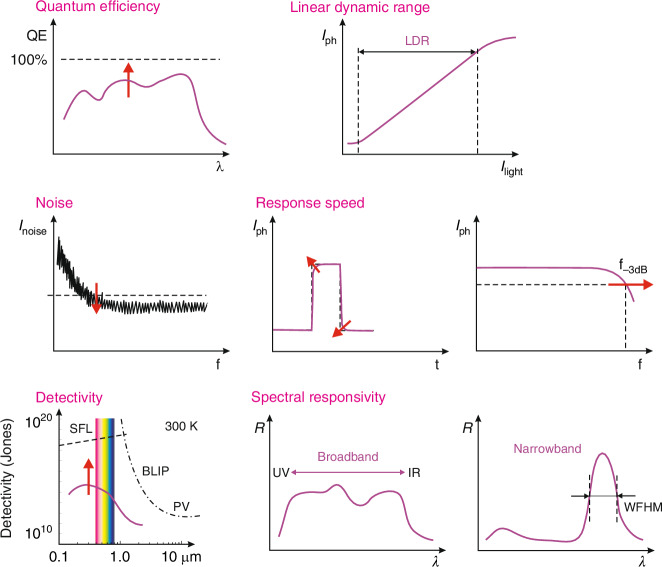
Challenges for emerging OPDs

The high-performance photosensitive OSCs have been fabricated, however efficient large areas *processing* has not been developed yet. The OSCs structural disorder and excitonic nature exhibit challenges related to the performance metrics needed to be addressed to compete with current ISCs technology. Figure 25 illustrates the required performance metrics for OSCs photodiodes.

Most organic materials are not suitable for mass production because they cannot withstand the high temperatures used for post-processing or they become unstable during long-time use at moderate temperatures. To overcome this challenge the researchers focused on modifying the photodetector’s buffer layer to improve stability, efficiency and detectivity. Improved performance can be achieved by stronger charge blocking and better hole extraction, as indicated by previous studies. Increasing the doping of interface layers is an effective way to achieve high OPD performance.

OSCs are more ecological and low-cost than ISC. Inherent electrical properties results in performance limitations to include low carriers mobility caused by the weak intermolecular interactions.

The OSC based photodetectors may operate in photoconductive, photovoltaic and FET photodetectors modes. They are mainly fabricated as single devices (photodiodes). The standard photodiodes are commonly used as pixels in 2D arrays, however, at the current stage of technology, the fabrication of OSC required for imaging arrays is still limited by yield. Reproducibility and scalability are also a challenge, as array production requires homogeneous pixels on a large scale.

The main message of this paper is to clarify the peculiarities of organic photodetectors when confronted with the typical conventional devices that dominate the global market. This objective was sought to be achieved by analyzing the fundamental physical properties of organic materials affecting photodetector performance, such as carrier mobilities, absorption coefficients and carrier lifetimes. With the current state of organic materials technology, these are no better than the properties of conventional semiconductors. Using the criterion $\sqrt{\alpha \tau }$, the high absorption coefficient (about 10^5 ^cm^‒1^) does not compensate for the lower values of carrier lifetimes compared to conventional Ge, Si and InGaAs type semiconductors (see Fig. 7).

The assessed utmost detectivity for OSC photodiodes is equivalent to typical ISCs photodiodes (see Fig. 10). The variation is close to three orders of magnitude. Detectivity > 10^14^ Jones was reported indicating on overestimates. The utmost OSC photodetectors performance, mainly phototransistors exceeding SFL/BLIP is caused by inaccurate assumption of parameters.

## Data Availability

All data generated or analysed during this study are included in this published article.

## References

[1] Peumans, P., Yakimov, A. & Forrest, S. R. Small molecular weight organic thin-film photodetectors and solar cells. *J. Appl. Phys.* **93**, 3693–3723 (2003).

[2] Forrest, S. R. The path to ubiquitous and low-cost organic electronic appliances on plastic. *Nature* **428**, 911–918 (2004). 15118718 10.1038/nature02498

[3] Shaheen, S. E., Ginley, D. S. & Jabbour, G. E. Organic based photovoltaics: toward low-cost power generation. *MRS Bull.* **30**, 10–19 (2005).

[4] Scott, J. C. & Bozano, L. D. Nonvolatile memory elements based on organic materials. *Adv. Mater.* **19**, 1452–1463 (2007).

[5] Xue, J. G. Perspectives on organic photovoltaics. *Polymer Rev.* **50**, 411–419 (2010).

[6] Baeg, K. J. et al. Organic light detectors: photodiodes and phototransistors. *Adv. Mater.* **25**, 4267–4295 (2013). 23483718 10.1002/adma.201204979

[7] Manousiadis, P. P. et al. Organic semiconductors for visible light communications. *Philos. Trans. A* **378**, 20190186 (2020). 10.1098/rsta.2019.0186 PMC7061996 32114909

[8] Simone, G. et al. Organic photodetectors and their application in large area and flexible image sensors: the role of dark current. *Adv. Funct. Mater.* **30**, 1904205 (2020).

[9] Forrest, S. R. *Organic Electronics: Foundations to Applications*, Vol. 1072 (Oxford: Oxford University Press, 2020).

[10] Ren, H. et al. Recent progress in organic photodetectors and their applications. *Adv. Sci.* **8**, 2002418 (2021). 10.1002/advs.202002418 PMC7788634 33437578

[11] Griffith, M. J. et al. Printable organic semiconductors for radiation detection: from fundamentals to fabrication and functionality. *Front. Phys.* **8**, 22 (2020).

[12] Pope, M. & Swenberg, C. E. *Electronic Processes in Organic Crystals and Polymers* 2nd edn. (Oxford: Oxford University Press, 1999).

[13] Bässler, H. & Köhler, A. Charge transport in organic semiconductors. In *Unimolecular and Supramolecular Electronics I* (ed Metzger, R. M.) 1−65 (Springer, 2012). 10.1007/128_2011_218 21972021

[14] Liu, C. et al. A unified understanding of charge transport in organic semiconductors: the importance of attenuated delocalization for the carriers. *Mater. Horizons* **4**, 608–618 (2017).

[15] Tang, C. G., Hou, K. Q. & Leong, W. L. The quest for air stability in organic semiconductors. *Chem. Mater.* **36**, 28–53 (2024).

[16] Lüssem, B., Riede, M. & Leo, K. Doping of organic semiconductors. *Phys. Status Solidi* **210**, 9–43 (2013).

[17] Scaccabarozzi, A. D. et al. Doping approaches for organic semiconductors. *Chem. Rev.* **122**, 4420–4492 (2022). 34793134 10.1021/acs.chemrev.1c00581

[18] Rogalski, A. et al. Infrared HOT photodetectors: status and outlook. *Sensors* **23**, 7564 (2023). 37688032 10.3390/s23177564 PMC10490682

[19] Rogalski, A. et al. Performance of low-dimensional solid room-temperature photodetectors—critical view. *Materials* **17**, 4522 (2024). 39336263 10.3390/ma17184522 PMC11433362

[20] Hutsch, S., Panhans, M. & Ortmann, F. Charge carrier mobilities of organic semiconductors: ab initio simulations with mode-specific treatment of molecular vibrations. *NPJ Comput. Mater.* **8**, 228 (2022).

[21] Klauk, H. Organic thin-film transistors. *Chem. Soc. Rev.* **39**, 2643–2666 (2010). 20396828 10.1039/b909902f

[22] Olivier, Y. et al. 25th Anniversary article: high-mobility hole and electron transport conjugated polymers: how structure defines function. *Adv. Mater.* **26**, 2119–2136 (2014). 24599835 10.1002/adma.201305809

[23] Krückemeier, L. et al. Developing design criteria for organic solar cells using well-absorbing non-fullerene acceptors. *Commun. Phys.* **1**, 27 (2018).

[24] Venkateshvaran, D. et al. Approaching disorder-free transport in high-mobility conjugated polymers. *Nature* **515**, 384–388 (2014). 25383522 10.1038/nature13854

[25] Rogalski, A. et al. Perovskite versus standard photodetectors. *Materials* **17**, 4029 (2024). 39203207 10.3390/ma17164029 PMC11356170

[26] Wu, J. Y. et al. A comparison of charge carrier dynamics in organic and perovskite solar cells. *Advanced Materials* **34**, 2101833 (2022). 34773315 10.1002/adma.202101833 PMC11469080

[27] Hendriks, K. H. et al. Small-bandgap semiconducting polymers with high near-infrared photoresponse. *J. Am. Chem. Soc.* **136**, 12130–12136 (2014). 25101518 10.1021/ja506265h

[28] Clarke, T. M. & Durrant, J. R. Charge photogeneration in organic solar cells. *Chem. Rev.* **110**, 6736–6767 (2010). 20063869 10.1021/cr900271s

[29] Kruse, P. W. The photon detection process. In *Optical and Infrared Detectors* (ed Keyes, R. J.) 5−69 (Springer, 1980)

[30] Kingston, R. H. *Detection of Optical and Infrared Radiation* 1st edn, Vol. 142 (Springer, 1978).

[31] Dereniak, E. L. & Boreman, G. D. *Infrared Detectors and Systems*. (Wiley, 1996).

[32] Wang, Y. Z. et al. Narrowband organic photodetectors – towards miniaturized, spectroscopic sensing. *Mater. Horizons* **9**, 220–251 (2022). 10.1039/d1mh01215k 34704585

[33] Tao, J. W. et al. Organic UV-sensitive phototransistors based on distriphenylamineethynylpyrene derivatives with ultra-high detectivity approaching 10^18^. *Adv. Mater.* **32**, 1907791 (2020). 10.1002/adma.201907791 32058647

[34] Song, I. et al. High-performance visible-blind UV phototransistors based on n-type naphthalene diimide nanomaterials. *ACS Appl. Mater. Interfaces* **10**, 11826–11836 (2018). 29560713 10.1021/acsami.8b01500

[35] Qi, Z. et al. High-performance thermally stable organic phototransistors based on PSeTPTI/PC_61_BM for visible and ultraviolet photodetection. *Adv. Funct. Mater.* **25**, 3138–3146 (2015).

[36] Han, T. et al. Ultrahigh photosensitive organic phototransistors by photoelectric dual control. *J. Mater. Chem. C* **7**, 4725–4732 (2019).

[37] Yang, B. et al. High performance ternary organic phototransistors with photoresponse up to 2600 nm at room temperature. *Adv. Funct. Mater.* **31**, 2103787 (2021).

[38] Li, X. et al. Background limited ultraviolet photodetectors of solar-blind ultraviolet detection. *Appl. Phys. Lett.* **103**, 171110 (2013).

[39] Bianconi, S., Lauhon, L. J. & Mohseni, H. Exaggerated sensitivity in photodetectors with internal gain. *Nat. Photonics* **15**, 714 (2021).

[40] Rogalski, A. Detectivities of WS_2_/HfS_2_ heterojunctions. *Nat. Nanotechnol.* **17**, 217–219 (2022). 35273361 10.1038/s41565-022-01076-6

[41] Rogalski, A. Overestimating the performance of photon ultraviolet detectors. *IEEE Electron Device Lett.* **44**, 805–808 (2023).

[42] Wang, F. et al. How to characterize figures of merit of two-dimensional photodetectors. *Nat. Commun.* **14**, 2224 (2023). 37076481 10.1038/s41467-023-37635-1 PMC10115793

[43] Fang, H. H. & Hu, W. D. Photogating in low dimensional photodetectors. *Adv. Sci.* **4**, 1700323 (2017). 10.1002/advs.201700323 PMC5737233 29270342

[44] Shan, T. et al. Organic photodiodes: device engineering and applications. *Front. Optoelectron.* **15**, 49 (2022). 36637681 10.1007/s12200-022-00049-w PMC9763529

[45] Pecunia, V., Natali, D. & Caironi, M. Organic photodetectors. In *Photodetectors: Materials, Devices and Applications* (ed Nabet, B.), 2^nd^ edition, 73−137 (Elsevier, 2023)

[46] Li, Y., Chen, H. & Zhang, J. H. Carrier blocking layer materials and application in organic photodetectors. *Nanomaterials* **11**, 1404 (2021). 34073349 10.3390/nano11061404 PMC8228918

[47] Rogalski, A. & Bielecki, Z. *Detection of Optical Signals* 1st edn, (CRC Press, 2022).

[48] Li, L. L. et al. Trap-assisted photomultiplication polymer photodetectors obtaining an external quantum efficiency of 37,500. *ACS Appl. Mater. Interfaces* **7**, 5890–5897 (2015). 25715745 10.1021/acsami.5b00041

[49] Park, S. et al. High mobility polymer based on a π-extended benzodithiophene and its application for fast switching transistor and high gain photoconductor. *Sci. Rep.* **4**, 5482 (2014). 24970637 10.1038/srep05482 PMC4073171

[50] Zhou, X. K. et al. Ultrahigh gain polymer photodetectors with spectral response from UV to near-infrared using ZnO nanoparticles as anode interfacial layer. *Adv. Funct. Mater.* **26**, 6619–6626 (2016).

[51] Sandberg, O. J. et al. Mid-gap trap state-mediated dark current in organic photodiodes. *Nat. Photonics* **17**, 368–374 (2023).

[52] Wang, J. B. et al. Broadband organic photodetectors based on ternary blend active layers with enhanced and spectrally flat response. *J. Mater. Chem. C* **8**, 14049–14055 (2020).

[53] Zhao, Z. et al. Recent progress on broadband organic photodetectors and their applications. *Laser Photon. Rev.* **14**, 2000262 (2020).

[54] Zhou, X. K., Yang, D. Z. & Ma, D. G. Extremely low dark current, high responsivity, all-polymer photodetectors with spectral response from 300 nm to 1000 nm. *Adv. Opt. Mater.* **3**, 1570–1576 (2015).

[55] Armin, A. et al. Narrowband light detection via internal quantum efficiency manipulation of organic photodiodes. *Nat. Commun.* **6**, 6343 (2015). 25721323 10.1038/ncomms7343

[56] Lan, Z. J. et al. Filter-free band-selective organic photodetectors. *Adv. Opt. Mater.* **8**, 2001388 (2020).

[57] Lan, Z. J. et al. Dual-band organic photodetectors for dual-channel optical communications. *Laser Photon. Rev.* **16**, 2100602 (2022).

[58] Xing, S. et al. Photomultiplication-type organic photodetectors for near-infrared sensing with high and bias-independent specific detectivity. *Adv. Sci.* **9**, 2105113 (2022). 10.1002/advs.202105113 PMC8895121 34994114

